# Harnack’s inequality for doubly nonlinear equations of slow diffusion type

**DOI:** 10.1007/s00526-021-02044-z

**Published:** 2021-08-27

**Authors:** Verena Bögelein, Andreas Heran, Leah Schätzler, Thomas Singer

**Affiliations:** 1 Fachbereich Mathematik, Universität Salzburg, Hellbrunner Str. 34, 5020 Salzburg, Austria; 2 Department of Data Science, Friedrich-Alexander-Universität Erlangen-Nürnberg, Cauerstrasse 11, 91058 Erlangen, Germany; 3 TeamBank AG, Beuthener Strasse 25, 90471 Nürnberg, Germany

**Keywords:** 35K55, 35K65, 35B45, 35B65

## Abstract

In this article we prove a Harnack inequality for non-negative weak solutions to doubly nonlinear parabolic equations of the form

$$\begin{aligned} \partial _t u - {{\,\mathrm{div}\,}}{\mathbf {A}}(x,t,u,Du^m) = {{\,\mathrm{div}\,}}F, \end{aligned}$$

where the vector field ${\mathbf {A}}$ fulfills *p*-ellipticity and growth conditions. We treat the slow diffusion case in its full range, i.e. all exponents $m > 0$ and $p>1$ with $m(p-1) > 1$ are included in our considerations.

## Introduction and results

Let $\Omega \subset {\mathbb {R}}^n$, $n \ge 2$, be a bounded open domain and (0, *T*) with $0<T<\infty$ a finite time interval. In the following, $\Omega _T := \Omega \times (0,T)$ denotes the related space-time cylinder. The prototype of the doubly nonlinear equations we are concerned with is

1.1 $$\begin{aligned} \partial _t u - {{\,\mathrm{div}\,}}\left( |Du^m|^{p-2}Du^m\right) = 0  \text { in }\Omega _T \end{aligned}$$

for non-negative solutions $u :\Omega _T \rightarrow {\mathbb {R}}_{\ge 0}$ with parameters $m \in (0,\infty )$ and $p \in (1,\infty )$. If $m=1$, (1.1) reduces to the parabolic *p*-Laplace equation, whereas for $p=2$ we retrieve the porous medium equation. Doubly nonlinear equations of type (1.1) are classified as doubly degenerate if $m > 1$ and $p >2$, singular-degenerate if $m > 1$ and $p \in (1,2)$, degenerate-singular if $m \in (0,1)$ and $p >2$ and doubly singular if $m \in (0,1)$ and $p \in (1,2)$. Furthermore, depending on the behavior of solutions, we distinguish between slow diffusion equations with $m(p-1) > 1$ and fast diffusion equations with $m(p-1) < 1$. The qualitative difference between both cases stems from the fact that in the former one solutions might have a compact support, while this is not possible in the latter one. In the present paper, we treat the complete slow diffusion range $p(m-1) > 1$, which includes the doubly degenerate case and the singular-degenerate and degenerate-singular slow diffusion case.

In the literature, (1.1) often appears in equivalent forms; cf. [17–20, 28, 33]. More precisely, we note that formally (1.1) is a transformation of

$$\begin{aligned} \partial _t u^{\widehat{m}} - {{\,\mathrm{div}\,}}\big (|Du|^{p-2}Du\big ) = 0 \end{aligned}$$

with $$\widehat{m} := \frac{1}{m}$$ and

$$\begin{aligned} \partial _t u - c(\ell ,p){{\,\mathrm{div}\,}}\big (u^{\ell } |Du|^{p-2}Du\big ) = 0 \end{aligned}$$

where $\ell := (m-1)(p-1)$. These representations of (1.1) can be shown to be equivalent. Let us also note that for $m>1$ there are two different notions of weak solutions to the porous medium equation and doubly nonlinear equations in the literature. The first one assumes that $u^{\frac{m+1}{2}}$ is weakly differentiable with respect to the space variable, whereas the second one claims this for $u^m$ (in the case $m<1$ only the latter one makes sense). For the prototype porous medium equation the equivalence of both notions of solutions has been shown in [6]. It is still an open problem if the same is true for doubly nonlinear equations and porous medium type equations with a general structure.

Harnack estimates play a crucial role in the regularity theory of partial differential equations. In the elliptic setting, essential contributions are due to Moser [25] for linear elliptic equations and Serrin [29] and Trudinger [31] for quasilinear elliptic equations. In the parabolic setting, the first results have been obtained by Hadamard [16] and Pini [27] for non-negative solutions of the heat equation. For the heat equation Harnack’s inequality takes the form

$$\begin{aligned} c^{-1} \sup _{B_\varrho \left( x_o\right) } u\left( \cdot ,t_o-\varrho ^2\right) \le u\left( x_o,t_o\right) \le c \sup _{B_\varrho \left( x_o\right) } u\left( \cdot ,t_o+\varrho ^2\right) \end{aligned}$$

with *waiting time* $\varrho ^2$. Moser [26] showed that this result is true for linear parabolic equations as well and demonstrated the necessity of the waiting time. Later, Trudinger [32] proved Harnack inequalities for quasilinear parabolic equations and the homogeneous doubly nonlinear equation

$$\begin{aligned} \partial _t \left( u^{p-1}\right) - {{\,\mathrm{div}\,}}\left( |Du|^{p-2}Du\right) = 0 \end{aligned}$$

with $p>1$. Using an approach based on mean value inequalities for suitable De Giorgi classes, Gianazza and Vespri [14] gave a proof that extends to more general operators *A*(*x*, *t*, *u*, *Du*) instead of $|Du|^{p-2}Du$. Finally, simplifying an approach originally introduced by Moser, Kinnunen & Kuusi [22] obtained Harnack’s inequality for the homogeneous doubly nonlinear equation, where the Lebesgue measure is replaced by a more general Borel measure. In the case of non-homogeneous nonlinear equations, the situation is more involved. DiBenedetto [7] proved that non-negative weak solutions of the parabolic *p*-Laplace equation and the porous medium equation satisfy an *intrinsic* Harnack inequality of the form

$$\begin{aligned} c^{-1} \sup _{B_{\varrho }\left( x_o\right) } u\left( \cdot ,t_o-t_w\right) \le u\left( x_o,t_o\right) \le c\inf _{B_\varrho \left( x_o\right) } u\left( \cdot ,t_o+t_w\right) \end{aligned}$$

with $t_w = c u(x_o,t_o)^{2-p} \varrho ^p$ for the parabolic *p*-Laplace equation and $t_w = c u(x_o,t_o)^{1-m} \varrho ^2$ for the porous medium equation. These Harnack inequalities are called intrinsic, because the waiting times depend on the solution itself. Loosely speaking, solutions of non-homogeneous equations behave like solutions of the heat equation in an intrinsic time scale. A counterexample [11] shows that a Harnack estimate with $t_w$ independent of *u* is false. Since the proof in [7] relies on comparison with explicit solutions, it cannot be adapted for general quasilinear equations. Nearly 20 years later, this problem was overcome by DiBenedetto, Gianazza & Vespri [9], whose proof only uses measure theoretical tools. The main novelty is the so-called *Expansion of Positivity*. The same method was used by Kuusi [23] to obtain weak Harnack estimates for super-solutions of nonlinear degenerate parabolic equations. For an extensive overview regarding the parabolic *p*-Laplace equation and the porous medium equation with the definition of weak solution involving $u^\frac{m+1}{2}$, we refer to the monograph [10] by DiBenedetto, Gianazza and Vespri and the survey [11] by Düzgün, Fornaro and Vespri. Harnack’s inequality for the prototype doubly nonlinear equation

1.2 $$\begin{aligned} \partial _t u - {{\,\mathrm{div}\,}}\left( |u|^{m-1} |Du|^{p-2} Du\right) = 0 \end{aligned}$$

has first been proved by Vespri [33] for the full range of parameters $p >1$ and $m+p > \max \{2,3-\frac{p}{n}\}$. The proof uses explicit constructions involving the Barenblatt solution and therefore cannot be applied to more general structures. For the doubly degenerate case Fornaro and Sosio [12] generalized the result to weak solutions of

$$\begin{aligned} \partial _t u - {{\,\mathrm{div}\,}}{\mathbf {A}}(x,t,u,Du) = {\mathbf {B}}(x,t,u,Du), \end{aligned}$$

where the operators ${\mathbf {A}}$ and ${\mathbf {B}}$ fulfill the conditions

$$\begin{aligned} \left\{ \begin{array}{rl} {\mathbf {A}}(x,t,u,\xi ) \cdot \xi &{}\ge c_0 \Phi (|u|) |\xi |^p - c^p, \\ |{\mathbf {A}}(x,t,u,\xi )| &{}\le c_1 \Phi (|u|) |\xi |^{p-1} + c^{p-1} \Phi (|u|)^\frac{1}{p}, \\ |{\mathbf {B}}(x,t,u,\xi )| &{}\le c_2 \Phi (|u|) |\xi |^{p-1} + c_2 c^{p-1} \Phi (|u|)^\frac{1}{p} \end{array} \right. \end{aligned}$$

with $p \ge 2$, positive constants $c_0, c_1, c_2, c$ and a function $\Phi$ satisfying an $(m-1)$-growth condition with $m \ge 1$. They used a definition of weak solution involving $u^\frac{m+1}{2}$. A weak Harnack inequality for super-solutions can be found in [24]. For the case of fast diffusion equations, we refer to the articles by Fornaro, Sosio and Vespri [13] and Vespri and Vestberg [34].

In this paper we prove Harnack’s inequality for the entire slow diffusion range and thereby close the gap for the by now missing singular-degenerate and degenerate-singular slow diffusion cases. Furthermore, we work with a definition of weak solution involving $u^m$, which is new even for the doubly degenerate case and the slow diffusion porous medium equation.

### Setting

We consider non-negative weak solutions to the doubly nonlinear equation

1.3 $$\begin{aligned} \partial _t u - {{\,\mathrm{div}\,}}{\mathbf {A}}\left( x,t,u,Du^m\right) = {{\,\mathrm{div}\,}}F  \text { in }\Omega _T \end{aligned}$$

with $m > 0$. For the vector field ${\mathbf {A}}:\Omega _T\times {\mathbb {R}}\times {\mathbb {R}}^{n} \rightarrow {\mathbb {R}}^{n}$ we assume that ${\mathbf {A}}$ is measurable with respect to $(x,t)\in \Omega _T$ for all $(u,\xi ) \in {\mathbb {R}}\times {\mathbb {R}}^{n}$ and continuous with respect to $(u,\xi )$ for a.e. $(x,t) \in \Omega _T$. Moreover, we assume that ${\mathbf {A}}$ satisfies the following growth and ellipticity conditions

1.4 $$\begin{aligned} {\mathbf {A}}(x,t,u,\xi ) \cdot \xi \ge \nu |\xi |^p  \text { and }  |{\mathbf {A}}(x,t,u,\xi )| \le L |\xi |^{p-1}, \end{aligned}$$

for $p>1$ and structure constants $0<\nu \le L<\infty$. We demand that

1.5 $$\begin{aligned} F \in L^\sigma (\Omega _T,{\mathbb {R}}^n) \end{aligned}$$

for some $\sigma >\frac{n+p}{p-1}$ and that the parameters *m* and *p* satisfy $m(p-1) > 1$ which means that we are in the slow diffusion range. In the following we abbreviate

$$\begin{aligned} d:= m(p-1)-1 > 0. \end{aligned}$$

We now give the precise definition of weak solution to (1.3) that we use throughout the paper.

#### Definition 1.1

Assume that the vector field ${\mathbf {A}}$ satisfies (1.4). A non-negative measurable function $u :\Omega _T \rightarrow {\mathbb {R}}_{\ge 0}$ in the class

$$\begin{aligned} u \in C^0\left( (0,T);L_{\mathrm {loc}}^{m+1}(\Omega )\right) \text { with } u^m \in L_{\mathrm {loc}}^p\left( 0,T;W_{\mathrm {loc}}^{1,p}(\Omega )\right) \end{aligned}$$

is a non-negative weak sub(super)-solution to the doubly nonlinear equation (1.3) if and only if the identity

1.6 $$\begin{aligned} \iint _{\Omega _T} \big [-u \cdot \partial _t \varphi + {\mathbf {A}}(x,t,u,Du^m) \cdot D\varphi \big ] \,\mathrm {d}x\mathrm {d}t \, \begin{matrix} (\ge ) \\ \le \end{matrix} \iint _{\Omega _T} F \cdot D\varphi \,\mathrm {d}x \mathrm {d}t \end{aligned}$$

holds true for any testing function $\varphi \in C_0^\infty (\Omega _T,{\mathbb {R}}_{\ge 0})$. If *u* is a weak sub- and super-solution it is called a weak solution.

We are now in the position to formulate the main result of our paper:

#### Theorem 1.2

Let $m >0$, $p>1$ with $m(p-1) > 1$ and *u* be a continuous, non-negative, weak solution to (1.3) in the sense of Definition 1.1, where the vector field ${\mathbf {A}}$ satisfies (1.4) and *F* satisfies (1.5). Moreover, let $(x_o,t_o)\in \Omega _T$ such that $u(x_o,t_o)>0$. Then, there exist constants $c_o,\gamma >1$ depending only on $n,m,p,L,\nu$ and $\sigma$ such that for all cylinders $B_{9\varrho }(x_o) \times (t_o - 4 \theta \varrho ^p,t_o + 4 \theta \varrho ^p) \Subset \Omega _T$, with

$$\begin{aligned} \theta = \left( \frac{c_o}{u(x_o,t_o)}\right) ^d\end{aligned}$$

we either have

1.7 $$\begin{aligned} \Vert F\Vert _{L^\sigma \left( \Omega _T\right) } \varrho ^{p-1-\frac{n+p}{\sigma }} \ge \tfrac{1}{\gamma }u\left( x_o,t_o\right) ^{d+ 1 -\frac{d}{\sigma }} \end{aligned}$$

or

1.8 $$\begin{aligned} \left( 2 \gamma ^2\right) ^{-1} \sup _{B_\varrho (x_o)} u\left( \cdot , t_o - (2 \gamma )^{-d} \theta \varrho ^p\right) \le u\left( x_o,t_o\right)&\le \gamma \inf _{B_{\varrho }(x_o)} u\left( \cdot ,t_o+\theta \varrho ^p\right) . \end{aligned}$$

Note that the continuity assumption in Theorem 1.2 is not restrictive. The Harnack inequality continues to hold for a.e. point $(x_o,t_o)\in \Omega _T$ if we state it for an arbitrary non-negative, weak solution to (1.3). However, for the sake of a neater exposition of the result, we prefer to state it for continuous solutions.

### Plan of the paper

In Sect. 2 we collect some auxiliary tools. Using $u^m - a^m$ for some level *a* as test function in (a mollified version of) the definition of weak sub- and super-solutions, we derive certain Caccioppoli inequalities in Sect. 3. For convenience of the reader we state all intermediate results for weak sub- respectively super-solutions instead of weak solutions, so that it becomes clear what the minimal assumptions are. Next, in Sect. 4 we show that weak sub-solutions to (1.3) are locally bounded and give a quantitative estimate. In Sect. 5 we prove so-called De Giorgi type lemmas. Loosely speaking, the first lemma shows that if a super-solution *u* to (1.3) is smaller than some level *M* only on a small enough proportion of a suitable cylinder, then *u* is larger than $\frac{M}{2}$ a.e. on a smaller cylinder contained in the first one. The second lemma gives an analogous statement for sub-solutions in the case that *u* is larger than a fixed level only on a small enough proportion of the bigger cylinder and consequently smaller than a fraction of the level on the smaller cylinder. The proofs of the statements rely in particular on the Caccioppoli estimates. In Sect. 6 we prove Expansion of Positivity of non-negative weak super-solutions. The conclusion of the section is that if

$$\begin{aligned} |\{u(t_o)\ge M\} \cap B_\varrho (x_o)| \ge \alpha |B_\varrho (x_o)| \end{aligned}$$

for a level $M>0$, $\alpha \in (0,1)$ and a suitable ball $B_\varrho (x_o)$, then $u \ge \kappa M$ a.e. in $B_{2\varrho } (x_o) \times \big (t_o + \frac{1}{2} b (\kappa M)^{-d}\varrho ^p,t_o + b (\kappa M)^{-d}\varrho ^p \big ]$. Here, the constants $b, \kappa \in (0,1)$ depend only on the data and $\alpha$. In the proof, the Caccioppoli estimates and the first De Giorgi type lemma are used. Finally, in Sect. 7 we deduce the intrinsic Harnack inequality stated in Theorem 1.2. To show the forward inequality, i.e. the second inequality in (1.8), after a transformation we use the second De Giorgi type lemma and iteratively apply Expansion of positivity. Subsequently, we prove that the forward inequality implies the backward Harnack inequality, i.e. the first inequality in (1.8). Actually, a more general version of the backward Harnack inequality is shown in Sect. 7.

## Preliminaries

### Notation

First, we introduce some notation used throughout the paper. For functions defined on $\Omega _T$, we denote the time slice at time $t\in (0,T)$ by $v(t) := v(\cdot ,t)$. For $z_o=(x_o,t_o)\in {\mathbb {R}}^n \times {\mathbb {R}}$ we define space-time cylinders

$$\begin{aligned} Q^-_{\varrho ,\theta }(z_o)&:= B_\varrho (x_o) \times \Lambda ^-_{\theta }(t_o) := B_\varrho (x_o) \times (t_o - \theta , t_o]\\ Q_{\varrho ,\theta }^+(z_o)&:= B_\varrho (x_o) \times \Lambda _{\theta }^+(t_o) := B_\varrho (x_o) \times (t_o , t_o + \theta ] \end{aligned}$$

with a radius $\varrho >0$ and time length $\theta > 0$ and let

$$\begin{aligned} Q_{\varrho ,\theta }(z_o) := Q^-_{\varrho ,\theta }(z_o) \cup Q^+_{\varrho ,\theta }(z_o) \end{aligned}$$

As usual, we let

$$\begin{aligned} (u-a)_+ := \max \{u-a,0\}, \qquad (u-a)_- := \max \{-(u-a),0\}, \end{aligned}$$

for $u,a\in {\mathbb {R}}$. Furthermore, for $u,a\ge 0$ we define the boundary term

2.1 $$\begin{aligned} {\mathfrak {b}}\left[ u^m,a^m\right] := \tfrac{m}{m+1}\left( a^{m+1}-u^{m+1}\right) - u\cdot \left( a^m - u^m\right) . \end{aligned}$$

### Mollification in time

Since weak solutions do not possess a time derivative in general we have to use mollification. To this end, for $v \in L^1(\Omega _T,{\mathbb {R}}^N)$ and $h>0$ we define the following mollification in time

2.2 $$\begin{aligned}{}[\![ v ]\!]_h := \tfrac{1}{h} \int _0^t e^{\frac{s-t}{h}} v(x,s) \,\mathrm {d}s, \end{aligned}$$

which formally satisfies the ordinary differential equation

2.3 $$\begin{aligned} \partial _t [\![v]\!]_h = -\tfrac{1}{h}\big ([\![v]\!]_h - v\big ) . \end{aligned}$$

Basic properties of $[\![ \cdot ]\!]_h$ are provided in the following lemma. For its proof and further information, we refer to [21, Lemma 2.2] and [5, Appendix B].

#### Lemma 2.1

Suppose that *X* is a separable Banach space. If $v \in L^r(0,T;X)$ for some $r \ge 1$, then the mollification $[\![ v ]\!]_h$ defined in (2.2) fulfills $[\![ v ]\!]_h \in L^r(0,T;X)$ and for any $t_o \in (0,T]$ there holds

$$\begin{aligned} \left\| [\![ v ]\!]_h\right\| _{L^r(0,t_o;X)} \le \left\| v\right\| _{L^r(0,t_o;X)}. \end{aligned}$$

Moreover, in the case $r<\infty$ we have $[\![ \cdot ]\!]_h \rightarrow v$ in $L^r(0,T;X)$ as $h \downarrow 0$.

Using the same technique as in [30, Lemma 3.6], we conclude that any sub(super)-solution to (1.3) in the sense of Definition 1.1 satisfies the mollified version of (1.6),

2.4 $$\begin{aligned}&\iint _{\Omega _T} \big [\partial _t [\![ u ]\!]_h \varphi + [\![ {\mathbf {A}}(x,t,u,Du^m) ]\!]_h \cdot D\varphi \big ] \,\mathrm {d}x\mathrm {d}t\nonumber \\&\quad \begin{matrix} (\ge ) \\ \le \end{matrix} \iint _{\Omega _T} [\![ F ]\!]_h \cdot D\varphi \,\mathrm {d}x\mathrm {d}t + \tfrac{1}{h} \int _\Omega u(0) \int _0^T e^{-\frac{s}{h}} \varphi \,\mathrm {d}s\mathrm {d}x \end{aligned}$$

for any $$\varphi \in C_0^\infty (\Omega _T,{\mathbb {R}}_{\ge 0})$$.

### Transformation

The following Lemma is an easy consequence of a change of variables.

#### Lemma 2.2

Let $T>0$, $I \subset {\mathbb {R}}$ be an open interval and $\Phi :I \rightarrow (0,T)$ an increasing $C^\infty$-diffeomorphism. Then, *u* is a weak sub(super)-solution to (1.3) associated to ${\mathbf {A}}$, *F* in $B_\varrho \times (0,T)$ if and only if the function $w(x,\tau ):=u(x,\Phi (\tau ))$ is a sub(super)-solution to (1.3) associated to the vector field

$$\begin{aligned} \widetilde{{\mathbf {A}}}(x,\tau ,u,\xi ) := \Phi '(\tau ){\mathbf {A}}\big (x,\Phi (\tau ),u,\xi \big ) \end{aligned}$$

and right-hand side ${\widetilde{F}}(x,\tau ) := \Phi '(\tau ) F(x,\Phi (\tau ))$ in $B_\varrho \times I$.

The next Lemma shows that the product of a non-negative weak super-solution *u* with a non-decreasing $C^1$-function $\gamma$ is a super-solution to a modified equation. A similar argument has already been used in [9].

#### Lemma 2.3

Let $\Omega \subset {\mathbb {R}}^n$ be bounded and open and $I\subset {\mathbb {R}}$ an open interval. Assume that *u* is a non-negative weak super-solution to (1.3) in $\Omega \times I$ associated to ${\mathbf {A}}$, *F* and $\gamma \in C^1(I)\cap C^0({\overline{I}})$ is non-decreasing and satisfies $\frac{1}{C} \le \gamma \le C$ on *I* for a constant $C\ge 1$. Then, the function ${\tilde{u}} := \gamma u$ is a non-negative weak super-solution to (1.3) in $\Omega \times I$ associated to the vector-field

$$\begin{aligned} \widetilde{{\mathbf {A}}}(x,t,u,\xi ) := \gamma (\tau ) {\mathbf {A}}\left( x,t,\tfrac{u}{\gamma (t)},\tfrac{\xi }{\gamma (t)^m}\right) \end{aligned}$$

and inhomogeneity $${\widetilde{F}}:=\gamma F$$.

#### Proof

In the following we abbreviate $\Omega _I:=\Omega \times I$. Let $\varphi \in C_0^\infty (\Omega _I,{\mathbb {R}}_{\ge 0})$. Then $\gamma \varphi \in C_0^1(\Omega _I,{\mathbb {R}}_{\ge 0})$. By assumption $\gamma '$, *u* and therefore also $[\![ u ]\!]_h$ are non-negative. By an approximation argument we may use $\gamma \varphi$ as testing function in the mollified weak formulation (2.4) on the interval *I* instead of (0, *T*). This leads to

$$\begin{aligned} -\iint _{\Omega _I} [\![u]\!]_h \gamma \partial _t \varphi \,\mathrm {d}x\mathrm {d}t&= \iint _{\Omega _I} \partial _t ([\![ u ]\!]_h \gamma ) \varphi \,\mathrm {d}x\mathrm {d}t= \iint _{\Omega _I} \big [\partial _t [\![ u ]\!]_h \gamma \varphi + [\![ u ]\!]_h \gamma ' \varphi \big ] \,\mathrm {d}x\mathrm {d}t\\&\ge \iint _{\Omega _I} \partial _t [\![ u ]\!]_h \gamma \varphi \,\mathrm {d}x\mathrm {d}t\\&\ge -\iint _{\Omega _I} [\![ {\mathbf {A}}(x,t,u,Du^m) ]\!]_h \cdot D (\gamma \varphi ) \,\mathrm {d}x\mathrm {d}t\\&\quad \, +\iint _{\Omega _I} [\![ F ]\!]_h \cdot D(\gamma \varphi ) \,\mathrm {d}x\mathrm {d}t+ \tfrac{1}{h} \int _\Omega u(0) \int _I e^{-\frac{s}{h}} (\gamma \varphi ) \,\mathrm {d}s\mathrm {d}x. \end{aligned}$$

Passing to the limit $h \downarrow 0$ with the help of Lemma 2.1 and taking into account that ${{\,\mathrm{spt}\,}}(\gamma \varphi )$ is compact in the last term on the right-hand side, this leads to

$$\begin{aligned} \iint _{\Omega _I} \left[ - \gamma u \partial _t \varphi + \gamma {\mathbf {A}}(x,t,u,Du^m) \cdot D\varphi \right] \,\mathrm {d}x\mathrm {d}t\ge \iint _{\Omega _I} \gamma F \cdot D\varphi \,\mathrm {d}x\mathrm {d}t\end{aligned}$$

for every $\varphi \in C_0^\infty (\Omega _I,{\mathbb {R}}_{\ge 0})$, which is in view of the definition of ${\tilde{u}}$ and ${\widetilde{F}}$ equivalent to

$$\begin{aligned} \iint _{\Omega _I} \left[ - {\tilde{u}}\partial _t \varphi + \gamma {\mathbf {A}}\left( x,t,\tfrac{\tilde{u}}{\gamma },\tfrac{D \tilde{u}^m}{\gamma ^m}\right) \cdot D \varphi \right] \,\mathrm {d}x\mathrm {d}t\ge \iint _{\Omega _I} {\widetilde{F}} \cdot D\varphi \,\mathrm {d}x\mathrm {d}t. \end{aligned}$$

Recalling the definition of $\widetilde{{\mathbf {A}}}$, this yields the claim. $\square$

Combining the last two lemmata leads to the following statement, which is used in the proof of the expansion of positivity.

#### Corollary 2.4

Let $T>0$ and *u* a non-negative weak super-solution to (1.3) in $B_\varrho \times (0,T)$ associated to ${\mathbf {A}}$ and *F*. Further, assume that $I \subset {\mathbb {R}}$ is an open interval, that $\Phi :I \rightarrow (0,T)$ is an increasing $C^\infty$-diffeomorphism and that $\gamma \in C^1(I)\cap C^0({\overline{I}})$ is non-decreasing and satisfies $\frac{1}{C} \le \gamma \le C$ on *I* for some constant $C\ge 1$. Then, the function $v(x,\tau ) := \gamma (\tau ) \cdot u(x,\Phi (\tau ))$ is a non-negative weak super-solution to (1.3) in $B_\varrho \times I$ associated to the vector-field

$$\begin{aligned} \widehat{{\mathbf {A}}}(x,\tau ,u,\xi ) := \gamma (\tau )\Phi '(\tau ) {\mathbf {A}} \Big (x,\Phi (\tau ),\tfrac{u}{\gamma (\tau )},\tfrac{\xi }{\gamma (\tau )^m}\Big ) \end{aligned}$$

and inhomogeneity $$\widehat{F}(x,\tau ) := \gamma (\tau )\Phi '(\tau )F(x,\Phi (\tau ))$$.

### Auxiliary lemmata

For a function $v \in W^{1,1}$ and $k<\ell$ the next lemma gives a local estimate for the product of the measures of superlevel sets $\{ v > \ell \}$ and sublevel sets $\{ v < k\}$ in terms of the $L^1$-norm of *Dv* on the intersection of their complements, cf. [8, Chap. I.2, Lemma 2.2 and Remark 2.3].

#### Lemma 2.5

Let $v\in W^{1,1}(B_\varrho (x_o))$ and $k,\ell \in {\mathbb {R}}$ with $k<\ell$. Then, there exists a constant *c* depending on *n* such that

$$\begin{aligned} (\ell -k) |B_\varrho (x_o) \cap \{v<k\}| \le \frac{c \varrho ^{n+1}}{|B_\varrho (x_o) \cap \{v>\ell \}|} \int _{B_\varrho (x_o) \cap \{k<v<\ell \}} |Dv| \,\mathrm {d}x . \end{aligned}$$

The following lemma can be found in the literature; cf. [1, Lemma 2.2] for $\alpha \in (0,1)$ and [15, inequality (2.4)] for $\alpha >1$.

#### Lemma 2.6

For any $\alpha >0$, there exists a constant $c=c(\alpha )$ such that, for all $a,b\ge 0$, the following inequality holds true:

$$\begin{aligned} \tfrac{1}{c}|b^\alpha - a^\alpha | \le (|a| + |b|)^{\alpha -1}|b-a| \le c |b^\alpha - a^\alpha |. \end{aligned}$$

The next lemma summarizes all properties we need concerning the boundary term ${\mathfrak {b}}$ defined in (2.1).

#### Lemma 2.7

Let $m>0$. There exists a constant $c=c(m)$ such that for every $u,a \ge 0$ we have

(i) $$\frac{1}{c} \big |u^\frac{m+1}{2} - a^\frac{m+1}{2}\big |^2 \le {\mathfrak {b}}[u^m,a^m] \le c \big |u^\frac{m+1}{2} - a^\frac{m+1}{2}\big |^2$$.
(ii) $$\frac{1}{c} |u^m-a^m|^2 \le (u+a)^{m-1}\, {\mathfrak {b}}[u^m,a^m] \le c |u^m-a^m|^2$$.

#### Proof

The proof of (i) can be found in [4, Lemma 2.3] for $m\ge 1$ and in [3, Lemma 3.4] for $0<m<1$. The inequalities in (ii) are a consequence of (i) and Lemma 2.6. $\square$

The following iteration lemma is a well known result and can be found for instance in [8, Chap. I.4, Lemma 4.1].

#### Lemma 2.8

Let $(Y_i)_{i \in {\mathbb {N}}_0}$ be a sequence of non-negative numbers satisfying

$$\begin{aligned} Y_{i+1} \le \kappa \, b^i Y_i^{1+\gamma } \qquad \text{ for } \text{ all }\,i \in {\mathbb {N}}_0 \end{aligned}$$

with some positive constants $\kappa , \gamma$ and $b >1$. If

$$\begin{aligned} Y_0 \le \kappa ^{-\frac{1}{\gamma }} b^{-\frac{1}{\gamma ^2}}, \end{aligned}$$

then $$Y_i \rightarrow 0$$ as $$i \rightarrow \infty $$.

Finally, we recall a parabolic version of the Gagliardo–Nirenberg inequality, see [8, Chapter I, Proposition 3.1] or [2, Lemma 3.1].

#### Lemma 2.9

Let $Q^-_{\varrho ,\theta }(z_o) \subset {\mathbb {R}}^{n+1}$ be a parabolic cylinder and $1<p,r<\infty$. For every

$$\begin{aligned} u \in L^\infty \left( t_o-\theta ,t_o;L^r\left( B_\varrho \left( x_o\right) \right) \right) \cap L^p\left( t_o-\theta ,t_o,W^{1,p}\left( B_\varrho \left( x_o\right) \right) \right) \end{aligned}$$

we have $u \in L^q(Q^-_{\varrho ,\theta }(z_o) )$ for $q=p(1+\frac{r}{n})$ with the estimate

$$\begin{aligned} \iint _{Q^-_{\varrho ,\theta }(z_o)}&|u|^q \,\mathrm {d}x\mathrm {d}t\\&\le c \bigg (\sup _{t\in (t_o-\theta ,t_o)} \int _{B_\varrho (x_o)\times \{t\}} |u|^r \,\mathrm {d}x\bigg )^{\frac{p}{n}} \int _{Q^-_{\varrho ,\theta }(z_o)} \left[ |Du|^p+\Big |\frac{u}{\varrho }\Big |^p \, \right] \,\mathrm {d}x\mathrm {d}t, \end{aligned}$$

where $$c=c(n,p,r)$$.

## Caccioppoli inequalities

In this section we derive energy estimates that are crucial in the course of the paper. We start with the energy estimates for weak super-solutions.

### Lemma 3.1

Let $m >0$, $p>1$ with $m(p-1) > 1$ and *u* be a non-negative weak super-solution to (1.3) in the sense of Definition 1.1, where the vector-field ${\mathbf {A}}$ fulfills the growth and ellipticity assumptions (1.4). Then, there exists a constant $c=c(p,\nu ,L)$ such that on any cylinder $Q^-_{\varrho ,\theta }(z_o) \Subset \Omega _T$ with $\varrho ,\theta > 0$, and for any $0<r<\varrho$, $0<s<\theta$ and $a\ge 0$ the following energy estimates

3.1 $$\begin{aligned}&\sup _{t \in \Lambda _s^-(t_o)} \int _{\{u<a\}\cap B_r(x_o)\times \{t\}} {\mathfrak {b}}[u^m,a^m] \,\mathrm {d}x+\iint _{\{ u<a \}\cap Q^-_{r,s}(z_o)} |D u^m|^p \,\mathrm {d}x\mathrm {d}t\nonumber \\&\quad \le c \iint _{\{ u < a\} \cap Q^-_{\varrho ,\theta }(z_o)} \bigg [\frac{{\mathfrak {b}}[u^m,a^m]}{\theta - s} + \frac{|u^m-a^m|^p}{(\varrho -r)^p} + |F|^\frac{p}{p-1}\bigg ] \,\mathrm {d}x\mathrm {d}t\end{aligned}$$

and

3.2 $$\begin{aligned}&\sup _{t \in \Lambda _s^-(t_o)} \int _{\{u<a \}\cap B_r(x_o)\times \{t\} } {\mathfrak {b}}\left[ u^m,a^m\right] \,\mathrm {d}x\le \int _{\{ u< a\} \cap B_\varrho (x_o)\times \{t_o-s\} } {\mathfrak {b}}\left[ u^m,a^m\right] \,\mathrm {d}x\nonumber \\&\quad +c \iint _{\{ u < a \} \cap Q^-_{\varrho ,\theta }(z_o)} \left[ \frac{|u^m-a^m|^p}{(\varrho -r)^p} + |F|^\frac{p}{p-1}\right] \,\mathrm {d}x\mathrm {d}t\end{aligned}$$

hold true, where ${\mathfrak {b}}[\cdot ,\cdot ]$ is defined in (2.1).

### Proof

Throughout the proof we abbreviate $Q^-_{\varrho ,\theta }\equiv Q^-_{\varrho ,\theta }(z_o)$ and $B_\varrho \equiv B_\varrho (x_o)$. Since the claimed estimates are local in nature, we may assume without loss of generality that $u \in C^0([0,T);L^{m+1}(\Omega ))$. An approximation argument shows that the mollified weak formulation (2.4) extends to non-negative testing functions $\varphi \in L^p(0,T;W^{1,p}_0(\Omega ))\cap L^{\frac{m+1}{m}}(\Omega _T)$ with compact support, since $[\![ u ]\!]_h\in C^0([0,T);L^{m+1}(\Omega ))$, $[\![ {\mathbf {A}}(x,t,u,Du^m) ]\!]_h$, $[\![ F ]\!]_h \in L^\frac{p}{p-1}(\Omega _T)$ and $u(0) \in L^{m+1}(\Omega )$ by the assumptions on *u*, growth condition (1.4) and Lemma 2.1. We therefore find that

3.3 $$\begin{aligned}&\iint _{\Omega _T} \Big [\partial _t [\![ u ]\!]_h \varphi + [\![ {\mathbf {A}}(x,t,u,Du^m) ]\!]_h \cdot D\varphi \Big ] \,\mathrm {d}x\mathrm {d}t\nonumber \\&\quad \ge \iint _{\Omega _T} [\![ F ]\!]_h \cdot D\varphi \,\mathrm {d}x\mathrm {d}t+\tfrac{1}{h} \int _\Omega u(0) \int _0^T e^{-\frac{s}{h}} \varphi \,\mathrm {d}s\mathrm {d}x\end{aligned}$$

holds true for any $\varphi \in L^p(0,T;W_0^{1,p}(\Omega ,{\mathbb {R}}_{\ge 0}))\cap L^{\frac{m+1}{m}}(\Omega _T)$ with compact support. For $\varepsilon >0$ and $t_1 \in \Lambda _s(t_o)=(t_o-s,t_o)$ we define cutoff functions $\eta \in W^{1,\infty }(B_\varrho (x_o),[0,1])$, $\zeta \in W^{1,\infty }(\Lambda _{\theta }(t_o),[0,1])$ and $\psi _\varepsilon \in W^{1,\infty }(\Lambda _{\theta }(t_o),[0,1])$ which satisfy

$$\begin{aligned} \eta (x)&= {\left\{ \begin{array}{ll} 1, &{} \text {for } x \in B_r(x_o),\\ 0, &{} \text {for } x \in \Omega \setminus B_\varrho (x_o), \end{array}\right. } \quad \text { and } |D\eta | \le \frac{2}{\varrho -r},\\ \zeta (t)&= {\left\{ \begin{array}{ll} 1, &{} \text {for } t \in (t_o - s, t_o+\theta ),\\ \frac{t-t_o + \theta }{\theta -s}, &{} \text {for } t \in (t_o - \theta , t_o - s), \end{array}\right. } \\ \psi _\varepsilon (t)&= {\left\{ \begin{array}{ll} 1, &{} \text {for } t \in (t_o - \theta , t_1],\\ 1-\frac{1}{\varepsilon }(t-t_1), &{} \text {for } t \in (t_1 , t_1 + \varepsilon ),\\ 0, &{} \text {for } t \in [t_1 + \varepsilon , t_o). \end{array}\right. } \end{aligned}$$

We choose

$$\begin{aligned} \varphi (x,t) = \eta ^p(x) \zeta (t) \psi _\varepsilon (t) \big (u^m(x,t) - a^m\big )_- \end{aligned}$$

as testing function in the mollified version (3.3) of the differential equation. For the first term on the left hand side we have

$$\begin{aligned}&\iint _{\Omega _T} \partial _t [\![ u ]\!]_h \varphi \,\mathrm {d}x\mathrm {d}t\\&\quad = -\iint _{\{u< a \}\cap Q^-_{\varrho ,\theta }} \eta ^p \zeta \psi _\varepsilon \partial _t [\![ u ]\!]_h \left( [\![u ]\!]_h^m - a^m\right) \,\mathrm {d}x\mathrm {d}t\\&\qquad -\iint _{\{ u<a\}\cap Q^-_{\varrho ,\theta }} \eta ^p \zeta \psi _\varepsilon \partial _t [\![ u ]\!]_h \big (u^m - [\![ u]\!]_h^m \big ) \,\mathrm {d}x\mathrm {d}t\\&\quad \le -\iint _{\{ u<a\}\cap Q^-_{\varrho ,\theta }} \eta ^p \zeta \psi _\varepsilon \partial _t [\![ u ]\!]_h \big ([\![ u]\!]_h^m - a^m\big ) \,\mathrm {d}x\mathrm {d}t\\&\quad = -\iint _{\{ u<a \}\cap Q^-_{\varrho ,\theta }} \eta ^p \zeta \psi _\varepsilon \partial _t \Big ( \tfrac{1}{m+1} [\![ u ]\!]_h^{m+1} + \tfrac{m}{m+1} a^{m+1} - a^m [\![ u ]\!]_h \Big ) \,\mathrm {d}x\mathrm {d}t\\&\quad = -\iint _{\{ u< a \}\cap Q^-_{\varrho ,\theta }} \eta ^p \zeta \psi _\varepsilon \partial _t {\mathfrak {b}}\big [ [\![ u ]\!]_h^m , a^m \big ] \,\mathrm {d}x\mathrm {d}t\\&\quad = \iint _{\{ u <a \}\cap Q^-_{\varrho ,\theta }} \eta ^p \big ( \zeta \psi _\varepsilon ' + \psi _\varepsilon \zeta ' \big ) {\mathfrak {b}}\big [ [\![ u]\!]_h^m , a^m \big ] \,\mathrm {d}x\mathrm {d}t, \end{aligned}$$

where we used in turn (2.3), the fact that $(u - [\![ u]\!]_h)(u^m - [\![ u]\!]_h^m ) \ge 0$ by monotonicity of $s \mapsto s^m$ and the definition of ${\mathfrak {b}}$. Since $[\![ u ]\!]_h \rightarrow u$ in $L_{\text {loc}}^{m+1}(\Omega _T)$ in the limit $h \downarrow 0$, we get

3.4 $$\begin{aligned}&\limsup _{h \downarrow 0} \iint _{\Omega _T} \partial _t [\![ u ]\!]_h \varphi \,\mathrm {d}x\mathrm {d}t\nonumber \\&\quad \le \iint _{\{u < a \}\cap Q^-_{\varrho ,\theta }} \eta ^p \big ( \zeta \psi _\varepsilon ' + \psi _\varepsilon \zeta ' \big ) {\mathfrak {b}}[ u^m , a^m ] \,\mathrm {d}x\mathrm {d}t=: \mathrm {I}_\varepsilon + \mathrm {II}_\varepsilon , \end{aligned}$$

where the meaning of $\mathrm {I}_\varepsilon$ and $\mathrm {II}_\varepsilon$ is clear in this context. We let $h\downarrow 0$ also in the diffusion term. For the resulting integral we use assumptions (1.4) and Young’s inequality to obtain

$$\begin{aligned}&\lim _{h \downarrow 0} \iint _{\Omega _T} [\![ {\mathbf {A}}(x,t,u,Du^m) ]\!]_h \cdot D\varphi \,\mathrm {d}x\mathrm {d}t\\&\quad = \iint _{\Omega _T} {\mathbf {A}}(x,t,u,Du^m) \cdot D\varphi \,\mathrm {d}x\mathrm {d}t\\&\quad = -\iint _{\{u<a \}\cap Q^-_{\varrho ,\theta }} {\mathbf {A}}(x,t,u,Du^m) \cdot D \big ( \eta ^p \zeta \psi _\varepsilon (u^m - a^m) \big ) \,\mathrm {d}x\mathrm {d}t\\&\quad = -\iint _{\{ u< a\}\cap Q^-_{\varrho ,\theta }} {\mathbf {A}}(x,t,u,Du^m) \cdot \big [\eta ^p \zeta \psi _\varepsilon Du^m + p\eta ^{p-1} \zeta \psi _\varepsilon (u^m - a^m) D\eta \big ] \,\mathrm {d}x\mathrm {d}t\\&\quad \le \iint _{\{u< a \}\cap Q^-_{\varrho ,\theta }} \Big [-\nu \eta ^p \zeta \psi _\varepsilon |D u^m|^p + pL\eta ^{p-1} \zeta \psi _\varepsilon |D\eta ||u^m - a^m| |Du^m|^{p-1}\Big ]\,\mathrm {d}x\mathrm {d}t\\&\quad \le -\tfrac{\nu }{2} \iint _{\{u< a\}\cap Q^-_{\varrho ,\theta }} \eta ^p \zeta \psi _\varepsilon |D u^m|^p \,\mathrm {d}x\mathrm {d}t+ c \iint _{\{u<a\}\cap Q^-_{\varrho ,\theta }} \frac{|u^m - a^m|^p}{(\varrho -r)^p} \,\mathrm {d}x\mathrm {d}t, \end{aligned}$$

where $c=c(p,\nu ,L)$. The second term on the right hand side of (3.3) vanishes in the limit $h \downarrow 0$, since $\varphi (0) \equiv 0$. In the first integral we pass to the limit $h \downarrow 0$ and then apply Young’s inequality. This yields

$$\begin{aligned}&\lim _{h \downarrow 0} \iint _{\Omega _T } [\![F]\!]_h \cdot D\varphi \,\mathrm {d}x\mathrm {d}t\\&\quad \ge -\int _{\{u< a\} \cap Q^-_{\varrho ,\theta }} \Big [\eta ^p \zeta \psi _\varepsilon |F| |Du^m| + |F| |u^m-a^m| |D\eta |\Big ] \,\mathrm {d}x\mathrm {d}t\\&\quad \ge -\int _{\{u< a\}\cap Q^-_{\varrho ,\theta } } \bigg [\tfrac{\nu }{4} \eta ^p \zeta \psi _\varepsilon |Du^m|^p +\frac{|u^m - a^m|^p}{(\varrho - r)^p} + c(p,\nu ) |F|^\frac{p}{p-1}\bigg ] \,\mathrm {d}x\mathrm {d}t. \end{aligned}$$

Inserting the preceding estimates into (3.3), we conclude that

$$\begin{aligned}&-\mathrm {I}_\varepsilon +\tfrac{\nu }{4} \iint _{\{ u< a\} \cap Q^-_{\varrho ,\theta }} \eta ^p \zeta \psi _\varepsilon |Du^m|^p \,\mathrm {d}x\mathrm {d}t\\&\quad \le \mathrm {II}_\varepsilon +c(p,\nu ,L) \iint _{\{u < a \} \cap Q^-_{\varrho ,\theta }} \bigg [\frac{|u^m - a^m|^p}{(\varrho -r)^p} + |F|^\frac{p}{p-1}\bigg ] \,\mathrm {d}x\mathrm {d}t. \end{aligned}$$

Now, we pass to the limit $\varepsilon \downarrow 0$ in the preceding inequality. Since $u \in C^0([0,T];L^{m+1}(\Omega ))$, for any $t_1 \in \Lambda _s(t_o)$ we obtainFurther, we have

$$\begin{aligned} \lim _{\varepsilon \downarrow 0} \iint _{\{u<a\} \cap Q^-_{\varrho ,\theta }} \eta ^p \zeta \psi _\varepsilon |Du^m|^p \,\mathrm {d}x\mathrm {d}t&\ge \iint _{\{u<a\} \cap B_r \times (t_o-s,t_1)} |Du^m|^p \,\mathrm {d}x\mathrm {d}t\end{aligned}$$

and

$$\begin{aligned} \mathrm {II}_\varepsilon \le \iint _{\{u < a \} \cap Q^-_{\varrho ,\theta }} \frac{{\mathfrak {b}}[u^m,a^m]}{\theta - s} \,\mathrm {d}x\mathrm {d}t. \end{aligned}$$

Altogether, we deduce the estimate

$$\begin{aligned}&\int _{\{u< a\} \cap B_r\times \{t_1\}} {\mathfrak {b}}[u^m,a^m] \,\mathrm {d}x+\tfrac{\nu }{4} \iint _{\{u<a\}\cap B_r \times (t_o-s,t_1)} |D u^m|^p \,\mathrm {d}x\mathrm {d}t\\&\quad \le c(p,\nu ,L) \iint _{\{ u <a\} \cap Q^-_{\varrho ,\theta }} \bigg [\frac{|u^m - a^m|^p}{(\varrho -r)^p} + \frac{{\mathfrak {b}}[u^m,a^m]}{\theta - s} + |F|^\frac{p}{p-1}\bigg ] \,\mathrm {d}x\mathrm {d}t\end{aligned}$$

for any $t_1 \in \Lambda _s(t_o)$. Finally, taking the supremum over $t_1 \in \Lambda _s(t_o)$ in the first term and passing to the limit $t_1 \uparrow t_o$ in the second term yields inequality (3.1).

In order to prove (3.2) we choose $\varphi (x,t) = \eta ^p(x) \psi _\varepsilon (t) (u^m(x,t) - a^m)_-$ as testing function in (3.3), where $\eta$ is defined as before and

$$\begin{aligned} \psi _\varepsilon (t)&= {\left\{ \begin{array}{ll} 0, &{} \text {for } t \in (t_o - \theta , t_1-\varepsilon ],\\ \frac{t-t_1+\varepsilon }{\varepsilon }, &{} \text {for } t \in (t_1-\varepsilon ,t_1),\\ 1, &{} \text {for } t \in [t_1, t_2],\\ \frac{t_2-t+\varepsilon }{\varepsilon }, &{} \text {for } t \in (t_2 , t_2 + \varepsilon ),\\ 0, &{} \text {for } t \in [t_2 + \varepsilon , t_o), \end{array}\right. } \end{aligned}$$

for $t_o-s \le t_1< t_2 < t_o$ and $\varepsilon >0$ small enough. The term involving the time derivative of $[\![u]\!]_h$ is treated as in (3.4). Thus, we find that

$$\begin{aligned}&\lim _{\varepsilon \downarrow 0} \bigg [ \limsup _{h \downarrow 0} \iint _{\Omega _T}\partial _t [\![ u ]\!]_h \varphi \,\mathrm {d}x\mathrm {d}t\bigg ] \\&\quad \le \lim _{\varepsilon \downarrow 0} \iint _{\{ u<a \} \cap Q^-_{\varrho ,\theta }} \eta ^p \psi _\varepsilon ' {\mathfrak {b}}[ u^m , a^m]\,\mathrm {d}x\mathrm {d}t\\&\quad = \int _{\{ u< a \} \cap B_\varrho \times \{t_1\}} \eta ^p {\mathfrak {b}}[ u^m , a^m] \,\mathrm {d}x-\int _{\{u< a\} \cap B_\varrho \times \{t_2\}} \eta ^p {\mathfrak {b}}[u^m , a^m]\,\mathrm {d}x\\&\quad \le \int _{\{u<a \} \cap B_\varrho \times \{t_1\}} {\mathfrak {b}}[ u^m , a^m] \,\mathrm {d}x-\int _{\{u <a \} \cap B_r\times \{t_2\}} {\mathfrak {b}}[ u^m , a^m] \,\mathrm {d}x\end{aligned}$$

for any $t_o-s \le t_1< t_2 < t_o$. For the diffusion term and the right side the same arguments as in the proof of (3.1) are applicable. Therefore by passing to the limits $h \downarrow 0$ and $\varepsilon \downarrow 0$ we obtain

$$\begin{aligned}&\int _{\{u<a \} \cap B_r\times \{t_2\}} {\mathfrak {b}}[ u^m , a^m] \,\mathrm {d}x+\tfrac{\nu }{2} \iint _{\{u<a\}\cap B_r \times (t_1,t_2)} |D u^m|^p \,\mathrm {d}x\mathrm {d}t\\&\quad \le \int _{\{u<a \} \cap B_\varrho \times \{t_1\}} {\mathfrak {b}}[ u^m , a^m] \,\mathrm {d}x\\&\qquad +c(p,\nu ,L) \iint _{\{ u < a \} \cap Q^-_{\varrho ,\theta }} \bigg [\frac{|u^m - a^m|^p}{(\varrho -r)^p} + |F|^\frac{p}{p-1} \bigg ] \,\mathrm {d}x\mathrm {d}t\end{aligned}$$

for any $t_o-s \le t_1< t_2 < t_o$. Omitting the second term on the left side, choosing $t_1 = t_o-s$ and taking the supremum over $t_2 \in \Lambda _s(t_o)$ leads to (3.2). $\square$

Similarly, we obtain energy estimates for sub-solutions. However, in the course of the paper we only need the analogue of (3.1).

### Lemma 3.2

Under the assumptions of Lemma 3.1 we obtain for any non-negative weak sub-solution to (1.3) the energy estimate

3.5 $$\begin{aligned}&\sup _{t \in \Lambda _s^-(t_o)} \int _{\{u>a \} \cap B_r(x_o)\times \{t\}} {\mathfrak {b}}[u^m,a^m] \,\mathrm {d}x+\iint _{\{ u>a \}\cap Q^-_{r,s}(z_o)} |D u^m|^p \,\mathrm {d}x\mathrm {d}t\nonumber \\&\quad \le c \iint _{\{ u > a\} \cap Q^-_{\varrho ,\theta }(z_o)} \bigg [\frac{{\mathfrak {b}}[u^m,a^m]}{\theta - s} + \frac{|u^m-a^m|^p}{(\varrho -r)^p} + |F|^\frac{p}{p-1}\bigg ] \,\mathrm {d}x\mathrm {d}t, \end{aligned}$$

for a constant $$c=c(p,\nu ,L)$$.

### Proof

The proof is analogous to the one of the energy estimate (3.1). Here, we choose the testing function

$$\begin{aligned} \varphi (x,t) = \eta ^p(x) \zeta (t) \psi _\varepsilon (t) \big (u^m(x,t) - a^m\big )_+ \end{aligned}$$

with the positive part of $u^m-a^m$ instead of the negative one. Similar arguments as in the proof of (3.1) then lead us to inequality (3.5). $\square$

## Local boundedness of non-negative weak sub-solutions

In this section we establish that non-negative weak sub-solutions to (1.3) are locally bounded. We argue by a parabolic version of De Giorgi classes.

### Theorem 4.1

Let $m>0$ and $p>1$ with $m(p-1) > 1$. Assume that *u* is a non-negative weak sub-solution to (1.3) in the sense of Definition 1.1 and $F \in L^\sigma (\Omega _T)$ with $\sigma > \frac{n+p}{p-1}$. Then *u* is locally bounded in $\Omega _T$ and for any cylinder $Q_0:=Q^-_{\varrho ,\theta }(z_o) \Subset \Omega _T$ with $0< \varrho ,\theta \le 1$ the quantitative estimate

$$\begin{aligned} \sup _{\frac{1}{2} Q_0} u \le c\, \big (\tfrac{1}{\varrho ^p}+\tfrac{1}{\theta }\big )^{\frac{n+p}{p(m+1)}} \Big [ \Vert u \Vert _{L^{mp}(Q_0)}^{\frac{mp}{m+1}} + \Vert F \Vert _{L^\sigma (Q_0)}^{\frac{\sigma }{m+1}} + 1 \Big ] \end{aligned}$$

holds true, where $\frac{1}{2} Q_0:= Q^-_{\frac{\varrho }{2},\frac{\theta }{2}}(z_o)$ and *c* is a constant depending on $n,m,p,\nu ,L$ and $\sigma$.

### Proof

Let $m' := \frac{m+1}{m}$ denote the conjugate Hölder exponent of $m+1$. For $i \in {\mathbb {N}}_0$ we define radii $\varrho _i$ and times $\theta _i$ by

$$\begin{aligned} \varrho _i := \tfrac{1}{2} \left( 1 + 2^{-i}\right) \varrho \quad \text { and }\quad \theta _i := \tfrac{1}{2} \left( 1 + 2^{-i}\right) \theta . \end{aligned}$$

Throughout the proof, we use the short-hand notation

$$\begin{aligned} Q_i := Q^-_{\varrho _i,\tau _i}(z_o)\subset Q_0. \end{aligned}$$

Furthermore, for a quantity $k \ge 1$ to be chosen later on, we consider levels

$$\begin{aligned} k_i := (1 - 2^{-i})^\frac{1}{m} k \end{aligned}$$

and the sequence of integrals

$$\begin{aligned} Y_i := \iint _{Q_i} \left( u^m - k_i^m\right) _+^p \,\mathrm {d}x\mathrm {d}t. \end{aligned}$$

Since $u^m \in L^p(\Omega _T)$ by definition, $Y_i$ is finite for any $i \in {\mathbb {N}}_0$. The idea of proof is to show a recursive estimate for $Y_i$. To this aim we first use Hölder’s inequality to obtain

4.1 $$\begin{aligned} Y_{i+1}&\le \left( \iint _{Q_{i+1}} \left( u^m - k_{i+1}^m\right) _+^{\frac{p(n+m')}{n}} \,\mathrm {d}x\mathrm {d}t\right) ^\frac{n}{n+m'} \big |\{u> k_{i+1}\} \cap Q_{i+1} \big |^{1-\frac{n}{n+m'}} \nonumber \\&=: \mathrm {I}^\frac{n}{n+m'} \cdot \big |\{u > k_{i+1}\} \cap Q_{i+1} \big |^{1-\frac{n}{n+m'}} , \end{aligned}$$

where the definition of $\mathrm {I}$ is clear in this context. First, by the Gagliardo–Nirenberg inequality from Lemma 2.9 we infer

$$\begin{aligned} \mathrm {I}&\le c \left[ \sup _{t \in \left( t_o-\theta _{i+1},t_o\right) } \int _{B_{\varrho _{i+1}}(x_o)\times \{t\}} \big (u^m - k_{i+1}^m\big )_+^\frac{m+1}{m} \,\mathrm {d}x\right] ^\frac{p}{n} \\&\quad \cdot \iint _{Q_{i+1}} \left[ \big |D\left( u^m-k_{i+1}^m\right) _+\big |^p + \frac{(u^m - k_{i+1}^m)_+^p}{\varrho ^p}\right] \,\mathrm {d}x\mathrm {d}t, \end{aligned}$$

for a constant $c = c(n,m,p)$. We now consider the integrand in the first integral on the right-hand side. For $u\ge k_{i+1}$ we have with the abbreviation

$$\begin{aligned} {\tilde{k}}_i^m := \tfrac{1}{2}\left( k_i^m + k_{i+1}^m\right) < k_{i+1} \end{aligned}$$

that

$$\begin{aligned} u^m + {\tilde{k}}_i^m \le 2u^m \le \frac{2k_{i+1}^m}{k_{i+1}^m - {\tilde{k}}_{i}^m}\left( u^m-{\tilde{k}}_i^m\right) \le 2^{i+3} \left( u^m-{\tilde{k}}_i^m\right) _+ \end{aligned}$$

and

$$\begin{aligned} u^m + {\tilde{k}}_i^m \ge u^m - {\tilde{k}}_i^m. \end{aligned}$$

Therefore, in view of Lemma 2.7 (ii) we obtain

$$\begin{aligned} \left( u^m - k_{i+1}^m\right) _+^\frac{m+1}{m}&= \left( u^m + {\tilde{k}}_{i}^m\right) _+^\frac{1-m}{m} \left( u^m + {\tilde{k}}_{i}^m\right) _+^\frac{m-1}{m} \left( u^m - k_{i+1}^m\right) _+^\frac{m+1}{m} \\&\le c\, 2^{\frac{(m-1)_+}{m} i} \left( u + {\tilde{k}}_{i}\right) _+^{1-m} \left( u^m - {\tilde{k}}_{i}^m\right) _+^\frac{m-1}{m} \left( u^m - k_{i+1}^m\right) _+^\frac{m+1}{m} \\&\le c\,2^{\frac{(m-1)_+}{m} i} \left( u + {\tilde{k}}_{i}\right) _+^{1-m} \left( u^m - {\tilde{k}}_{i}^m\right) _+^2 \\&\le c\,2^{\frac{(m-1)_+}{m} i} {\mathfrak {b}}\left[ u^m, {\tilde{k}}_{i}^m\right] \chi _{\left\{ u>{\tilde{k}}_i\right\} }. \end{aligned}$$

Using this inequality above and applying the Caccioppoli inequality (3.5) from Lemma 3.2, yields

$$\begin{aligned} \mathrm {I}&\le c \left[ \sup _{t \in (t_o-\theta _{i+1},t_o)} \int _{\{u>{\tilde{k}}_{i}\}\cap B_{\varrho _{i+1}}(x_o)\times \{t\}} {\mathfrak {b}}[u^m, {\tilde{k}}_{i}^m] \,\mathrm {d}x\right] ^\frac{p}{n} \\&\quad \cdot \iint _{\{u> {\tilde{k}}_{i}\}\cap Q_{i+1}} \left[ |Du^m|^p + \frac{\left( u^m - {\tilde{k}}_{i}^m\right) ^p}{\varrho ^{p}}\right] \,\mathrm {d}x\mathrm {d}t\\&\le c \left[ \iint _{\{u > {\tilde{k}}_{i}\}\cap Q_i} \left[ \frac{2^{\frac{(m-1)_+}{m} i} {\mathfrak {b}}\left[ u^m, {\tilde{k}}_{i}^m\right] }{\theta _i-\theta _{i+1}} + \frac{\left( u^m - {\tilde{k}}_{i}^m\right) ^p}{\left( \varrho _i-\varrho _{i+1}\right) ^p} + |F|^\frac{p}{p-1}\right] \,\mathrm {d}x\mathrm {d}t\right] ^{\frac{n+p}{n}} , \end{aligned}$$

for a constant $c = c(n,m,p,\nu ,L)$. For $u>{\tilde{k}}_i$ we now estimate the ${\mathfrak {b}}$-term with the help of Lemma 2.7 (i), the assumption $m+1\le mp$ and the fact that $k_i<{\tilde{k}}_{i} < k$ with $k \ge 1$. In this way we obtain

$$\begin{aligned} {\mathfrak {b}}[u^m, {\tilde{k}}_{i}^m]&\le \big |u^\frac{m+1}{2} - {\tilde{k}}_{i}^\frac{m+1}{2}\big |^2 \le 2u^{m+1} \le 2\left( u^{mp} + 1\right) \\&\le c\left[ \left( u^m - {\tilde{k}}_{i}^m\right) ^p + k^{mp}\right] = c\left[ \left( u^m - {\tilde{k}}_{i}^m\right) ^p + 2^{(i+2)p}\left( {\tilde{k}}_i^m-k_i^m\right) ^{p}\right] \\&\le c\,2^{ip} \big (u^m - k_{i}^m\big )^p, \end{aligned}$$

with $c=c(p)$, so that

$$\begin{aligned} \mathrm {I}&\le c \left[ \iint _{\{u> {\tilde{k}}_{i}\}\cap Q_i} \left[ 2^{i \left( p+ \frac{(m-1)_+}{m} \right) } \left( \tfrac{1}{\varrho ^p} + \tfrac{1}{\theta } \right) \left( u^m - k_{i}^m\right) ^p + |F|^\frac{p}{p-1}\right] \,\mathrm {d}x\mathrm {d}t\right] ^{\frac{n+p}{n}} \\&\le c\, \left[ 2^{i \left( p + \frac{(m-1)_+}{m} \right) } \left( \tfrac{1}{\varrho ^p} + \tfrac{1}{\theta } \right) Y_i + \Vert F \Vert _{L^\sigma (Q_0)}^{\frac{p}{p-1}} \big |\{u > {\tilde{k}}_{i}\}\cap Q_i\big |^{1-\frac{p}{\sigma (p-1)}} \right] ^{\frac{n+p}{n}} , \end{aligned}$$

where $c = c(n,m,p,\nu ,L)$. Further, we have that

$$\begin{aligned} \big |\{u> {\tilde{k}}_{i}\}\cap Q_i\big | \big ({\tilde{k}}_{i}^m - k_i^m\big )_+^p \le \iint _{\{u > {\tilde{k}}_{i}\}\cap Q_i} \left( u^m - k_i^m\right) _+^p \,\mathrm {d}x\mathrm {d}t\le Y_i, \end{aligned}$$

which together with $k \ge 1$ implies that

4.2 $$\begin{aligned} \big |\{u > {\tilde{k}}_{i}\}\cap Q_i\big | \le \frac{2^{(i+2)p}}{k^{mp}} Y_i \le 2^{(i+2)p} Y_i. \end{aligned}$$

Finally, the preceding computations together with $0<\varrho \le 1$ and $Y_i \le \Vert u\Vert _{L^{mp}(Q_0)}^{mp}$ lead to

$$\begin{aligned} \mathrm {I}&\le c \left[ 2^{i \left( p+ \frac{(m-1)_+}{m} \right) } \big (\tfrac{1}{\varrho ^p} + \tfrac{1}{\theta } \big ) \Big (\Vert u \Vert _{L^{mp}(Q_0)}^\frac{mp^2}{\sigma (p-1)} + \Vert F \Vert _{L^\sigma (Q_0)}^{\frac{p}{p-1}} \Big ) Y_i^{1-\frac{p}{\sigma (p-1)}} \right] ^\frac{n+p}{n}, \end{aligned}$$

with a constant $c=c(n,m,p,\nu ,L)$. Inserting this inequality into (4.1) and using (4.2), we conclude that

$$\begin{aligned} Y_{i+1}&\le c \bigg [2^{i \left( p+ \frac{(m-1)_+}{m} \right) } \big (\tfrac{1}{\varrho ^p} + \tfrac{1}{\theta } \big ) \Big (\Vert u \Vert _{L^{mp}(Q_0)}^\frac{mp}{\sigma } + \Vert F \Vert _{L^\sigma (Q_0)} \Big )^{\frac{p}{p-1}} Y_i^{1-\frac{p}{\sigma (p-1)}} \bigg ]^\frac{n+p}{n+m'} \\&\quad \cdot \bigg [\frac{2^{ip}}{k^{mp}} Y_i\bigg ]^\frac{m'}{n+m'} \\&\le \kappa b^i Y_i^{1+\gamma }, \end{aligned}$$

where we used the abbreviations

$$\begin{aligned} \kappa&:= \frac{c\big (\tfrac{1}{\varrho ^p}+\tfrac{1}{\theta }\big )^\frac{n+p}{n+m'}}{k^{\frac{p(m+1)}{n+m'}}} \Big (\Vert u \Vert _{L^{mp}(Q_0)}^\frac{mp}{\sigma } + \Vert F \Vert _{L^\sigma (Q_0)} \Big )^\frac{p(n+p)}{(p-1)(n+m')}, \\ b&:= 2^{\frac{\left( p+ \frac{(m-1)_+}{m} \right) (n+p)+pm'}{n+m'}}, \\ \gamma&:= \tfrac{p}{n+m'} \big (1 - \tfrac{n+p}{\sigma (p-1)}\big ). \end{aligned}$$

Since $\sigma > \frac{n+p}{p-1}$, we have that $\gamma >0$. Choosing $k \ge 1$ large enough, such that

$$\begin{aligned} k \ge c \big (\tfrac{1}{\varrho ^p} + \tfrac{1}{\theta }\big )^{\frac{n+p}{p(m+1)}} \ \Vert u \Vert _{L^{mp}(Q_0)}^{\frac{mp}{m+1} \left( 1 - \frac{n+p}{\sigma (p-1)} \right) } \Big (\Vert u \Vert _{L^{mp}(Q_0)}^\frac{mp}{\sigma } + \Vert F \Vert _{L^\sigma (Q_0)} \Big )^\frac{n+p}{(p-1)(m+1)} \end{aligned}$$

with a suitable constant $c=c(n,m,p,\nu ,L,\sigma )$, we find that

$$\begin{aligned} Y_0 = \iint _{Q_0} u^{mp} \,\mathrm {d}x\mathrm {d}t\le \kappa ^{-\frac{1}{\gamma }} b^{-\frac{1}{\gamma ^2}}. \end{aligned}$$

Thus, the assumptions of Lemma 2.8 are satisfied. Consequently we find that $Y_i \rightarrow 0$ as $i \rightarrow \infty$, which implies $u\le k$ a.e. in $\frac{1}{2} Q_0$. The claim of the theorem now follows by an application of Young’s inequality. $\square$

## De Giorgi type lemmas

In this section we will prove certain De Giorgi type lemmata for weak sub- and super-solutions. We start with the one for super-solutions.

### Lemma 5.1

Let $m >0$, $p>1$ with $m(p-1) > 1$ and *u* be a bounded non-negative weak super-solution to (1.3) in the sense of Definition 1.1, where the vector-field ${\mathbf {A}}$ satisfies (1.4) and $F \in L^\sigma (\Omega _T)$ for some $\sigma >\frac{n+p}{p-1}$. Moreover, consider $z_o\in \Omega _T$ and $\varrho ,\theta ,M>0$ such that

$$\begin{aligned} Q^-_{2\varrho ,2^p\tau }(z_o)\Subset \Omega _T, \qquad \text{ where }\, \tau :=\theta M^{-d} \varrho ^p. \end{aligned}$$

Then, there exists $\nu _1\in (0,1)$ depending only on $n,m,p,\nu ,L,\sigma$ and $\theta$, such that: If

5.1 $$\begin{aligned}&\big |\{ u<M\}\cap Q^-_{2\varrho ,2^p\tau }(z_o)\big | \le \nu _1 |Q^-_{2\varrho ,2^p\tau }(z_o)|\nonumber \\&\quad \Vert F\Vert _{L^\sigma (\Omega _T)} \le \big (\tfrac{M^{m}}{\varrho }\big )^{p-1} |Q^-_{\varrho ,\tau }(z_o)|^{\frac{1}{\sigma }} , \end{aligned}$$

then

$$\begin{aligned} u \ge \tfrac{M}{2} \quad \text { a.e.~in } Q^-_{\varrho ,\tau }(z_o) \end{aligned}$$

holds true.

### Proof

For $i \in {\mathbb {N}}_0$ define radii $\varrho _i$ and times $\tau _i$ by

$$\begin{aligned} \varrho _i:= \varrho \left( 1+2^{-i}\right) \quad \text { and }\quad \tau _i := \theta M^{-d}\varrho _i^p \end{aligned}$$

as well as levels

$$\begin{aligned} k_i :=\tfrac{M}{2} \left( 1+2^{-i}\right) . \end{aligned}$$

To shorten notation, we introduce

$$\begin{aligned} Q_i := Q^-_{\varrho _i,\tau _i}\left( z_o\right) , \qquad A_i := \{u<k_i\}\cap Q_i \quad \text { and } \quad Y_i:=\frac{|A_i|}{|Q_i|} \le 1. \end{aligned}$$

At this stage, we use the Caccioppoli inequality (3.1). Since $0 \le u < k_i$ on $A_i$ and $\frac{M}{2}\le k_i\le M$ and by Lemma 2.7 (ii), we estimate the term involving ${\mathfrak {b}}$ on the left-hand side by

$$\begin{aligned} {\mathfrak {b}}\left[ u^m,k_i^m\right] \ge \tfrac{1}{c(m)} \left( k_i + u\right) ^{1-m} \left( u^m - k_i^m\right) _-^2 \ge \tfrac{1}{c(m)} M^{1-m} \left( u^m - k_i^m\right) _-^2 , \end{aligned}$$

while for the one on the right-hand side we obtain by Lemma 2.7 (i) that

$$\begin{aligned} {\mathfrak {b}}\left[ u^m,k_i^m\right] \le c(m) \left( u^{\frac{m+1}{2}}-k_i^{\frac{m+1}{2}}\right) _-^2. \end{aligned}$$

Thus, we conclude that

$$\begin{aligned}&\sup _{t\in \left( t_o-\tau _{i+1},t_o\right) } \int _{B_{\varrho _{i+1}}\times \{t\}} M^{1-m} \left( u^m-k_i^m\right) _-^2 \,\mathrm {d}x+ \iint _{Q_{i+1}} |D\left( u^m-k_i^m\right) _-|^p \,\mathrm {d}x\mathrm {d}t \\&\quad \le c \iint _{A_i} \left[ 2^{(i+1)p}\frac{\left( u^m-k_i^m\right) _-^p}{\varrho ^p} +2^{ip}M^d\frac{\left( u^{\frac{m+1}{2}}-k_i^{\frac{m+1}{2}}\right) _-^2}{\theta \varrho ^p}+ |F|^\frac{p}{p-1} \right] \,\mathrm {d}x\mathrm {d}t \\&\quad \le c\, 2^{ip}\left[ \frac{M^{mp}}{\varrho ^p}\left( 1+\tfrac{1}{\theta }\right) |A_i|^{\frac{p}{\sigma (p-1)}} + \Vert F\Vert _{L^\sigma (\Omega _T)}^\frac{p}{p-1} \right] |A_i|^{1-\frac{p}{\sigma (p-1)}} \\&\quad \le \frac{c\, 2^{ip}M^{mp}}{\varrho ^p}\left[ \big (1+\tfrac{1}{\theta }\big ) |A_i|^{\frac{p}{\sigma (p-1)}} + |Q_i|^{\frac{p}{\sigma (p-1)}} \right] |A_i|^{1-\frac{p}{\sigma (p-1)}} \\&\quad \le \frac{c\, 2^{ip}M^{mp}}{\varrho ^p} |Q_i|^{\frac{p}{\sigma (p-1)}} |A_i|^{1-\frac{p}{\sigma (p-1)}}, \end{aligned}$$

where in the second last line we used assumption (5.1). Note that $c=c(m,p,\nu ,L,\theta )$. Next, we use Hölder’s inequality with exponents $\frac{n+2}{n}$ and $\frac{n+2}{2}$, the Gagliardo–Nirenberg inequality from Lemma 2.9 with $r=2$ and *p* and the preceding estimate. This leads to

$$\begin{aligned}&\iint _{A_{i+1}} (u^m-k_i^m)_-^p \,\mathrm {d}x\mathrm {d}t \\&\quad \le \left( \iint _{A_{i+1}} \left( u^m-k_i^m\right) ^{\frac{p(n+2)}{n}}_- \,\mathrm {d}x\mathrm {d}t\right) ^{\frac{n}{n+2}} |A_i|^{\frac{2}{n+2}} \\&\quad \le c \left( \sup _{t\in \left( t_o-\tau _{i+1},t_o\right) } \int _{B_{\varrho _{i+1}}\times \{t\}} \left( u^m-k_i^m\right) _-^2 \,\mathrm {d}x \right) ^{\frac{p}{n+2}}\\&\quad \cdot \left( \iint _{Q_{i+1}} |D\left( u^m-k_i^m\right) _-|^p +\frac{\left( u^m-k_i^m\right) ^p_-}{\varrho _{i+1}^p} \,\mathrm {d}x\mathrm {d}t \right) ^{\frac{n}{n+2}}|A_i|^{\frac{2}{n+2}} \\&\quad \le c\, 2^{ip\frac{n+p}{n+2}} M^\frac{p(m-1)}{n+2} \left[ \frac{M^{mp}}{\varrho ^p} |Q_i|^{\frac{p}{\sigma (p-1)}} |A_i|^{-\frac{p}{\sigma (p-1)}} \right] ^\frac{n+p}{n+2} |A_i|^{1+\frac{p}{n+2}} \end{aligned}$$

with a constant $c=c(n,m,p,\nu ,L)$. Moreover, due to Lemma 2.6 we have

$$\begin{aligned} \left( k_{i}^m-k_{i+1}^m\right)&= \big (\tfrac{M}{2} \big )^m \left( (1+2^{-i})^m-(1+2^{-(i+1)})^m \right) \\&\ge \tfrac{1}{c(m)} \left( \tfrac{M}{2} \right) ^m \left( 2+2^{-i}+2^{-(i+1)}\right) ^{m-1}2^{-(i+1)} \ge \tfrac{1}{c(m)}\, 2^{-i} M^m, \end{aligned}$$

so that

$$\begin{aligned} \iint _{A_{i+1}} \left( u^m-k_i^m\right) _-^p \,\mathrm {d}x\mathrm {d}t \ge \left( k_{i}^m-k_{i+1}^m\right) ^p|A_{i+1}| \ge \tfrac{1}{c(m,p)}\, 2^{-ip} M^{mp} |A_{i+1}|. \end{aligned}$$

Combing the preceding estimates yields

$$\begin{aligned} |A_{i+1}| \le c\, 2^{ip \left( 1 + \frac{n+p}{n+2}\right) } M^{\frac{p(m-1)}{n+2} - mp} \left[ \frac{M^{mp}}{\varrho ^p} |Q_i|^{\frac{p}{\sigma (p-1)}} |A_i|^{-\frac{p}{\sigma (p-1)}} \right] ^\frac{n+p}{n+2} |A_i|^{1+\frac{p}{n+2}} \end{aligned}$$

with a constant $c=c(n,m,p,\nu ,L,\theta )$. Dividing the above inequality by $|Q_{i+1}|$, using the fact that $\frac{|Q_i|}{|Q_{i+1}|} = c(n,p)$ shows that

$$\begin{aligned} Y_{i+1}&\le c\, 2^{ip \left( 1 + \frac{n+p}{n+2}\right) } M^{\frac{p(m-1)}{n+2} - mp} |Q_i|^{\frac{p}{n+2}} \left[ \frac{M^{mp}}{\varrho ^p} \right] ^\frac{n+p}{n+2} Y_i^{1 + \frac{p}{n+2} - \frac{p(n+p)}{\sigma (n+2)(p-1)}} \\&\le c\, 2^{ip \left( 1 + \frac{n+p}{n+2}\right) } Y_i^{1 + \frac{p}{n+2} - \frac{p(n+p)}{\sigma (n+2)(p-1)}}, \end{aligned}$$

where *c* depends only on $n,m,p,\nu ,L,\theta$. This brings us into the position to apply Lemma 2.8 with $\kappa = c$, $b = 2^{p \left( m + \frac{n+p}{n+2}\right) }$ and $\gamma = \frac{p}{n+2} - \frac{p(n+p)}{\sigma (n+2)(p-1)}>0$ (since $\sigma > \frac{n+p}{p-1}$), where $\nu _1\in (0,1)$ can be chosen in dependence on the data. This shows $Y_i \rightarrow 0$ as $i\rightarrow \infty$, which yields the claim. $\square$

Now we turn our attention to the De Giorgi type lemma for sub-solutions.

### Lemma 5.2

Let $m >0$, $p>1$ with $m(p-1) > 1$ and *u* be a bounded non-negative weak sub-solution to (1.3) in the sense of Definition 1.1, where the vector-field ${\mathbf {A}}$ satisfies (1.4) and $F \in L^\sigma (\Omega _T)$ for some $\sigma >\frac{n+p}{p-1}$. Moreover, consider $z_o\in \Omega _T$, $\varrho , \theta ,M, \mu _+>0$ and $a,\zeta \in (0,1)$, such that

$$\begin{aligned} Q^-_{2\varrho ,2^p\tau }(z_o)\Subset \Omega _T, \qquad \text{ where }\, \tau :=\theta M^{-d} \varrho ^p \end{aligned}$$

and

5.2 $$\begin{aligned} \sup _{Q^-_{2\varrho ,2^p\tau }(z_o)} u \le M \le \mu _+. \end{aligned}$$

Then, there exists $\nu _2\in (0,1)$ depending only on $n,m,p,\nu ,L,\sigma ,\theta ,a$ and $\zeta$ such that: If

5.3 $$\begin{aligned} \big |\big \{u^m\ge \mu _+^m-\zeta M^m\big \}\cap Q^-_{2\varrho ,2^p\tau }(z_o)\big | \le \nu _2 |Q^-_{2\varrho ,2^p\tau }(z_o)| \end{aligned}$$

and

5.4 $$\begin{aligned} \Vert F\Vert _{L^\sigma (\Omega _T)} \le \big (\tfrac{M^{m}}{\varrho }\big )^{p-1} |Q^-_{\varrho ,\tau }(z_o)|^{\frac{1}{\sigma }} , \end{aligned}$$

then

5.5 $$\begin{aligned} u^m \le \mu _+^m -a\zeta M^m \quad \text { a.e.~in } Q^-_{\varrho ,\tau }(z_o). \end{aligned}$$

### Proof

As before, we define for $i\in {\mathbb {N}}_0$

$$\begin{aligned} \varrho _i := \varrho \left( 1+2^{-i}\right) \quad \text { and } \quad \tau _i :=\theta M^{-d}\varrho _i^p \end{aligned}$$

as well as levels

$$\begin{aligned} k_i^m := \mu _+^m-\big (\tfrac{1-a}{2^i} + a \big )\zeta M^m \end{aligned}$$

and sets

$$\begin{aligned} Q_i:=Q^-_{\varrho _i,\tau _i}(z_o) \quad \text { and } \quad A_i:= \{u > k_i\}\cap Q_i. \end{aligned}$$

In the following we will apply the Caccioppoli inequality (3.5) from Lemma 3.2. Using the definition of $A_i$ and Lemma 2.7 (i), (ii) and the fact that $\frac{1}{c(\zeta )}M\le k_i\le u\le M$ on $A_i$, we estimate the terms involving ${\mathfrak {b}}$ by

$$\begin{aligned} \tfrac{1}{c(m,\zeta )} M^{1-m} \left( u^m-k_i^m\right) _+^2&\le \left( k_i + u\right) ^{1-m} \left( u^m - k_i^m\right) _+^2 \\&\le c(m) {\mathfrak {b}}\left[ u^m,k_i^m\right] \\&\le c(m) \left( u^{\frac{m+1}{2}} - k_i^{\frac{m+1}{2}}\right) _+^2. \end{aligned}$$

Thus, by the Caccioppoli inequality (3.5) and assumption (5.4), we obtain

$$\begin{aligned}&\sup _{t\in \left( t_o-\tau _{i+1},t_o\right) } \int _{B_{\varrho _{i+1}}\times \{t\}} M^{1-m} \left( u^m - k_i^m\right) _+^2 \,\mathrm {d}x+ \iint _{Q_{i+1}} |D\left( u^m-k_i^m\right) _+|^p \,\mathrm {d}x\mathrm {d}t \\&\quad \le c \iint _{A_i} \left[ 2^{(i+1)p} \frac{\left( u^m-k_i^m\right) _+^p}{\varrho ^p} +2^{ip}M^{d} \frac{\left( u^{\frac{m+1}{2}} - k_i^{\frac{m+1}{2}}\right) _+^2}{\theta \varrho ^p} +|F|^\frac{p}{p-1} \right] \,\mathrm {d}x\mathrm {d}t \\&\quad \le c\, 2^{ip}\left[ \frac{M^{mp}}{\varrho ^p} + \Vert F\Vert _{L^\sigma (\Omega _T)}^\frac{p}{p-1} |A_i|^{-\frac{p}{\sigma (p-1)}} \right] |A_i| \\&\quad \le \frac{c\, 2^{ip}M^{mp}}{\varrho ^p} |Q_i|^{\frac{p}{\sigma (p-1)}} |A_i|^{1-\frac{p}{\sigma (p-1)}}, \end{aligned}$$

for a constant $c=c(m,p,\nu ,L,\theta ,\zeta )$. Similarly as before, we use Hölder’s inequality, the Gagliardo–Nirenberg inequality from Lemma 2.9 with $r=2$ and *p* and the last estimate to conclude

$$\begin{aligned}&\iint _{A_{i+1}} \left( u^m-k_i^m\right) _+^p \,\mathrm {d}x\mathrm {d}t \\&\quad \le \left( \iint _{A_{i+1}} \left( u^m-k_i^m\right) ^{\frac{p(n+2)}{n}}_+ \,\mathrm {d}x\mathrm {d}t \right) ^{\frac{n}{n+2}} |A_i|^{\frac{2}{n+2}} \\&\quad \le c \left( \sup _{t\in \left( t_o-\tau _{i+1},t_o\right) } \int _{B_{\varrho _{i+1}}\times \{t\}} \left( u^m-k_i^m\right) _+^2 \,\mathrm {d}x \right) ^{\frac{p}{n+2}}\\&\quad \cdot \left( \iint _{Q_{i+1}} |D(u^m-k_i^m)_+|^p +\frac{\left( u^m-k_i^m\right) ^p_+}{\varrho _{i+1}^p} \,\mathrm {d}x\mathrm {d}t \right) ^{\frac{n}{n+2}} |A_i|^{\frac{2}{n+2}} \\&\le c\, 2^{ip \frac{n+p}{n+2}} M^\frac{p(m-1)}{n+2} \left[ \frac{M^{mp}}{\varrho ^p} |Q_i|^{\frac{p}{\sigma (p-1)}} |A_i|^{-\frac{p}{\sigma (p-1)}} \right] ^\frac{n+p}{n+2} |A_i|^{1+\frac{p}{n+2}} \end{aligned}$$

for a constant $c=c(n,m,p,\nu ,L,\theta ,\zeta )$. Notice that

$$\begin{aligned} \iint _{A_{i+1}} \left( u^m-k_i^m\right) _+^p \,\mathrm {d}x\mathrm {d}t \ge \left( k_{i+1}^m-k_i^m\right) ^p |A_{i+1}| = 2^{-(i+1)p} (1-a)^p \zeta ^p M^{mp} |A_{i+1}|. \end{aligned}$$

Combining the preceding two estimates leads to

$$\begin{aligned} |A_{i+1}| \le c\, 2^{ip \left( 1+\frac{n+p}{n+2}\right) } M^{\frac{p(m-1)}{n+2} - mp} \left[ \frac{M^{mp}}{\varrho ^p} |Q_i|^{\frac{p}{\sigma (p-1)}} |A_i|^{-\frac{p}{\sigma (p-1)}} \right] ^\frac{n+p}{n+2} |A_i|^{1+\frac{p}{n+2}}, \end{aligned}$$

with a constant $c=c(n,m,p,\nu ,L,\theta ,a,\zeta )$. By completely the same reasoning as in the proof of Lemma 5.1 we infer that $Y_i \rightarrow 0$ as $i\rightarrow \infty$, provided we choose $\nu _2\in (0,1)$ small enough in dependence on the data. $\square$

## Expansion of positivity

In this section, we prove the so called Expansion of Positivity of a non-negative weak super-solution *u*. The Expansion of Positivity is crucial in the proof of Harnack’s inequality. In a first step we show the following lemma, which ensures a certain propagation of positivity in measure.

### Lemma 6.1

Let $m >0$, $p>1$ with $m(p-1) > 1$ and *u* a non-negative weak super-solution to (1.3), and let $\alpha \in (0,1]$ and $M>0$. Then, there exist $\varepsilon = \varepsilon (m,\alpha ) \in (0,1)$ and $\delta = \delta (m,p,\nu ,L,\alpha ) \in (0,1)$ such that the following holds: Whenever $z_o=(x_o,t_o)\in \Omega _T$ and $\varrho >0$ such that $Q^+_{\varrho ,\delta M^{-d}\varrho ^p}(z_o)\subset \Omega _T$ and

6.1 $$\begin{aligned} \big |\{u(t_o)\ge M\}\cap B_\varrho (x_o)\big | \ge \alpha |B_\varrho (x_o)| \end{aligned}$$

and

6.2 $$\begin{aligned} \Vert F\Vert _{L^\sigma (\Omega _T)} \le \left( \tfrac{M^{m}}{\varrho }\right) ^{p-1} |Q^+_{\varrho ,\delta M^{-d}\varrho ^p}(z_o)|^{\frac{1}{\sigma }} , \end{aligned}$$

are satisfied, then

6.3 $$\begin{aligned} \big |\{u(t) \ge \varepsilon M\}\cap B_\varrho (x_o)\big | \ge \tfrac{\alpha }{2} |B_\varrho (x_o)| \quad \text { for all } t \in [ t_o,t_o+\delta M^{-d} \varrho ^p ) . \end{aligned}$$

### Proof

In the following we abbreviate $Q_0:= Q^+_{\varrho ,\delta M^{-d}\varrho ^p}(z_o)$ with $\delta \in (0,1)$ to be chosen later. The idea of the proof is to show that if (6.1) and (6.2) are valid, then

$$\begin{aligned} \big |\{ u(t) < \varepsilon M \}\cap B_{\varrho }(x_o)\big | \le \left( 1-\tfrac{\alpha }{2}\right) |B_\varrho (x_o)| \end{aligned}$$

holds true for all $t \in [ t_o,t_o+\delta M^{-d} \varrho ^p )$, which is equivalent to (6.3). Therefore in a first step we let $s \in (0,1)$ and compute

6.4 $$\begin{aligned}&|\{ u(t)< \varepsilon M \}\cap B_{\varrho }(x_o)| \nonumber \\&\quad \le |\{ u(t)< \varepsilon M \}\cap B_{(1-s)\varrho }(x_o)| + |B_{\varrho }(x_o) \setminus B_{(1-s)\varrho }(x_o)| \nonumber \\&\quad \le |\{ u(t) < \varepsilon M \}\cap B_{(1-s)\varrho }(x_o)| + ns |B_{\varrho }(x_o)|. \end{aligned}$$

To estimate the first term on the right hand side we use the Caccioppoli inequality (3.2) from Lemma 3.1. Taking $r=(1-s)\varrho$ and $a=M$ leads to

$$\begin{aligned}&\int _{\{u< M\}\cap B_{(1-s)\varrho }(x_o)\times \{t\}} {\mathfrak {b}}[u^m,M^m] \,\mathrm {d}x\\&\quad \le \int _{\{ u< M \}\cap B_\varrho (x_o)\times \{t_o\}} {\mathfrak {b}}[u^m,M^m] \,\mathrm {d}x+ c \iint _{\{ u< M \}\cap Q_0} \frac{|u^m - M^m|^p}{(s\varrho )^p} \,\mathrm {d}x\mathrm {d}t \\&\quad + c\iint _{\{ u < M \} \cap Q_0} |F|^\frac{p}{p-1} \,\mathrm {d}x\mathrm {d}t\\&\quad =: \mathrm {I} + \mathrm {II} + \mathrm {III} \end{aligned}$$

for any $t \in [t_o,t_o+\delta M^{-d} (1-s)^p\varrho ^p)$ with a constant $c=c(p,\nu ,L)$. Recalling the definition of the boundary term ${\mathfrak {b}}$ from (2.1) we estimate the left hand side by

$$\begin{aligned}&\int _{\{u< M\}\cap B_{(1-s)\varrho }(x_o)\times \{t\}} {\mathfrak {b}}[u^m,M^m] \,\mathrm {d}x\\&\quad \ge \int _{\{ u< \varepsilon M \}\cap B_{(1-s)\varrho }(x_o)\times \{t\}} {\mathfrak {b}}[u^m,M^m] \,\mathrm {d}x\\&\quad = \int _{\{ u< \varepsilon M \}\cap B_{(1-s)\varrho }(x_o)\times \{t\}} \tfrac{m}{m+1} M^{m+1} - M^m u +\underbrace{\tfrac{1}{m+1} u^{m+1} }_{\ge 0} \,\mathrm {d}x\\&\quad \ge \int _{\{ u< \varepsilon M \}\cap B_{(1-s)\varrho }(x_o)\times \{t\}} \tfrac{m}{m+1} M^{m+1} - \varepsilon M^{m+1} \,\mathrm {d}x\\&\quad \ge \tfrac{m}{m+1} M^{m+1} \left( 1-\varepsilon \tfrac{m+1}{m}\right) \big |\{ u(t) < \varepsilon M \}\cap B_{(1-s)\varrho }(x_o)\big | \end{aligned}$$

for $\varepsilon \in (0,\frac{m}{m+1})$ to be chosen later. For the first term on the right-hand side, we use again the definition of ${\mathfrak {b}}$ and assumption (6.1) to obtain

$$\begin{aligned} \mathrm {I}&= \int _{\{ u< M \}\cap B_\varrho (x_o)\times \{t_o\}} \tfrac{m}{m+1}M^{m+1} \underbrace{- M^m u + \tfrac{1}{m+1}u^{m+1}}_{\le 0} \,\mathrm {d}x\\&\le \tfrac{m}{m+1} M^{m+1} |\{u(t_o)< M\}\cap B_\varrho (x_o)| \\&\le \tfrac{m}{m+1} M^{m+1} (1-\alpha ) |B_\varrho (x_o)|. \end{aligned}$$

Further, we have that

$$\begin{aligned} \mathrm {II}&\le c M^{mp} (s\varrho )^{-p} |Q_0| = c\,\delta s^{-p} M^{m+1} |B_\varrho (x_o)| \end{aligned}$$

and in view of assumption that (6.2) we obtain

$$\begin{aligned} \mathrm {III}&\le c\Vert F\Vert _{L^\sigma (\Omega _T)}^\frac{p}{p-1} |Q_0|^{1-\frac{p}{\sigma (p-1)}} \le c \big (\tfrac{M^{m}}{\varrho }\big )^{p} |Q_0| = c\,\delta M^{m+1} |B_\varrho (x_o)|. \end{aligned}$$

Altogether this leads to

$$\begin{aligned}&\tfrac{m}{m+1} M^{m+1} \left( 1-\varepsilon \tfrac{m+1}{m}\right) \big |\{ u(t) < \varepsilon M \}\cap B_{(1-s)\varrho }(x_o)\big | \\&\quad \le \left[ \tfrac{m}{m+1} M^{m+1} (1-\alpha ) + c M^{m+1} \delta \left( s^{-p}+1\right) \right] |B_\varrho (x_o)| , \end{aligned}$$

which is the same as

$$\begin{aligned} \big |\{ u(t) < \varepsilon M \}\cap B_{(1-s)\varrho }(x_o)\big | \le \frac{1}{1-\varepsilon \tfrac{m+1}{m}} \left[ 1 - \alpha + c \delta \tfrac{m+1}{m}(s^{-p} + 1) \right] |B_\varrho (x_o)|. \end{aligned}$$

Combining the last estimate with (6.4), and taking into account that $0<1-\varepsilon \tfrac{m+1}{m} < 1$, we get

$$\begin{aligned} \big |\{ u(t) < \varepsilon M \}\cap B_{\varrho }(x_o)\big | \le \frac{1}{1-\varepsilon \tfrac{m+1}{m}} \left[ 1 - \alpha + c \delta \tfrac{m+1}{m} \left( s^{-p} + 1\right) + ns \right] |B_\varrho (x_o)| \end{aligned}$$

for any $t \in [ t_o,t_o+\delta M^{-d} \varrho ^p )$ with $c=c(p,\nu ,L)$. Now we choose $s=\frac{\alpha }{8n} \in (0,1)$ and thereafter $\delta =\delta (m,p,\nu ,L,\alpha )$ small enough to ensure $c \delta \frac{m+1}{m} (s^{-p} + 1) \le \frac{\alpha }{8}$. This leads to

$$\begin{aligned} \big |\{ u(t) < \varepsilon M \}\cap B_{\varrho }(x_o)\big | \le \frac{1}{1-\varepsilon \tfrac{m+1}{m}} \left( 1 - \tfrac{3 \alpha }{4} \right) |B_\varrho (x_o)| \end{aligned}$$

for all $t \in [ t_o,t_o+\delta M^{-d} \varrho ^p )$. Choosing

$$\begin{aligned} \varepsilon \le \frac{m}{m+1} \left( 1- \frac{1-\frac{3\alpha }{4}}{1-\frac{\alpha }{2}} \right) \in (0,1), \end{aligned}$$

we conclude the proof. $\square$

### Remark 6.2

From the proof of Lemma 6.1 we observe that $\varepsilon$ and $\delta$ are monotonically increasing with respect to $\alpha$.

The preceding lemma at hand, we are now able to prove the Expansion of Positivity for non-negative weak super-solutions to the doubly degenerate equation (1.3).

### Proposition 6.3

(Expansion of Positivity) Let $m >0$, $p>1$ with $m(p-1) > 1$ and *u* be a non-negative weak super-solution to (1.3). For fixed $\alpha \in (0,1]$ there exist constants $b, \kappa \in (0,1)$ and $c\ge 1$ depending only on *n*, *m*, *p*, $\nu$, *L*, $\sigma$ and $\alpha$ such that the following holds true: We consider $z_o=(x_o,t_o)\in \Omega _T$, $M>0$ and

6.5 $$\begin{aligned} \varrho \in (0,\varrho _0], \qquad \text{ where } \varrho _0 := \min \left\{ \tfrac{1}{8}{{\,\mathrm{dist}\,}}\left( x_o,\partial \Omega \right) , \left[ \frac{(T-t_o)(\kappa M)^d}{b}\right] ^{\frac{1}{p}}\right\} . \end{aligned}$$

Supposed that

6.6 $$\begin{aligned} \big |\{u(t_o)\ge M\} \cap B_\varrho (x_o)\big | \ge \alpha |B_\varrho (x_o)| \end{aligned}$$

and

6.7 $$\begin{aligned} \Vert F\Vert _{L^\sigma (\Omega _T)} \le \tfrac{1}{c} \left( \tfrac{M^{m}}{\varrho }\right) ^{p-1} \big |Q^+_{\varrho , M^{-d}\varrho ^p}(z_o)\big |^{\frac{1}{\sigma }} , \end{aligned}$$

are satisfied, then we have

$$\begin{aligned} u \ge \kappa M \qquad \text {a.e.\,in } B_{2\varrho } (x_o) \times \big (t_o + \tfrac{1}{2} b (\kappa M)^{-d}\varrho ^p, t_o + b (\kappa M)^{-d}\varrho ^p \big ]. \end{aligned}$$

### Proof

The proof of Proposition 6.3 is divided into several steps. Throughout the proof we denote by $\varepsilon = \varepsilon (m,\alpha ) \in (0,1)$ and $\delta = \delta (m,p,\nu ,L,\alpha ) \in (0,1)$ the constants from Lemma 6.1.

### Application of lemma 6.1

For $j_\star \in {\mathbb {N}}$ to be chosen later in dependence on *n*, *m*, *p*, $\nu$, *L*, $\sigma$ and $\alpha$ we define

$$\begin{aligned} \varrho _0 := \min \big \{\tfrac{1}{8}{{\,\mathrm{dist}\,}}(x_o,\partial \Omega ), \varrho _1\big \}, \quad \text{ where } \varrho _1 := \left[ \frac{(T-t_o) M^{d}}{\delta } \exp \bigg (-\frac{2^{2p+j_\star d}}{\delta \varepsilon ^{d}}\bigg ) \right] ^\frac{1}{p} \end{aligned}$$

and

$$\begin{aligned} s_0 := \frac{1}{M} \left( \frac{\delta \varrho _1^p}{T-t_o} \right) ^{\frac{1}{d}} = \exp \bigg (-\frac{2^{2p+j_\star d}}{\delta d\varepsilon ^{d}}\bigg ) < 1. \end{aligned}$$

Note that

$$\begin{aligned} B_{8\varrho }(x_o) \times \big [t_o,t_o+\delta (sM)^{-d}\varrho ^p\big ) \subset \Omega _T \qquad \text{ for } \text{ all }\, \varrho \in (0,\varrho _0] \,\text{ and } \,s \in [s_0,1]. \end{aligned}$$

Now we fix $\varrho \in (0,\varrho _0]$ and assume that (6.6) is satisfied and that

6.8 $$\begin{aligned} \Vert F \Vert _{L^\sigma \left( \Omega _T\right) } \le \left( \tfrac{(s_0M)^{m}}{\varrho }\right) ^{p-1} \big |Q^+_{\varrho , \delta M^{-d}\varrho ^p}(z_o)\big |^{\frac{1}{\sigma }}. \end{aligned}$$

Then, the assumptions of Lemma 6.1 are fulfilled with *M* replaced by *sM* for any $s \in [s_0,1]$. Thus, we find that

$$\begin{aligned} \big |\{ u(t)\ge \varepsilon s M \}\cap B_\varrho (x_o)\big | \ge \tfrac{\alpha }{2} |B_\varrho (x_o)| \end{aligned}$$

for all $s\in [s_0,1]$ and all $t \in [t_o,t_o+\delta (sM)^{-d} \varrho ^p)$.

### Transforming to another problem

For $\tau \ge 0$ we let $s(\tau ) := e^{-\frac{\tau }{d}}$. Then, we have $s(\tau ) \in [s_0,1]$ for $\tau \in [0,\tau _0]$, where

$$\begin{aligned} \tau _0 := d\ln \left( \tfrac{1}{s_0}\right) = \tfrac{2^{2p+j_\star d}}{\delta \varepsilon ^{d}}. \end{aligned}$$

Next, we define

$$\begin{aligned} {\widetilde{\Phi }}(\tau ) := \delta \big ( s(\tau ) M \big )^{-d} \varrho ^p = \delta M^{-d} \varrho ^p e^\tau \qquad \text{ for }\, \tau \in [0,\tau _0]. \end{aligned}$$

From Step 6.1 we deduce that for any $\tau \in [0,\tau _0]$ there holds

$$\begin{aligned} \big [t_o,t_o+{\widetilde{\Phi }} (\tau ) \big ) \subset (0,T) \end{aligned}$$

and

$$\begin{aligned} \big |\{ u(t) \ge \varepsilon s(\tau ) M \}\cap B_\varrho (x_o)\big | \ge \tfrac{\alpha }{2} |B_\varrho (x_o)| \qquad \text{ for } \text{ all }\, t\in \big [t_o,t_o+{\widetilde{\Phi }} (\tau )\big ). \end{aligned}$$

In particular, letting

$$\begin{aligned} \Phi (\tau ) := t_o+{\widetilde{\Phi }}(\tau ) \qquad \text{ for }\, \tau \in [0,\tau _0], \end{aligned}$$

we have that

6.9 $$\begin{aligned} \big |\big \{ u(\Phi (\tau )) \ge \varepsilon s(\tau ) M \big \}\cap B_\varrho (x_o) \big | \ge \tfrac{\alpha }{2} |B_\varrho (x_o)| \qquad \text{ for } \text{ all }\, \tau \in [0,\tau _0]. \end{aligned}$$

Finally, we let $\gamma (\tau ) := {\widetilde{\Phi }}(\tau )^\frac{1}{d}$. Then, Corollary 2.4 ensures that

$$\begin{aligned} v(x,\tau ) := \gamma (\tau ) u(x,\Phi (\tau )) \end{aligned}$$

is a non-negative weak super-solution to

$$\begin{aligned} \partial _t v - {{\,\mathrm{div}\,}}\widehat{{\mathbf {A}}}\big (x,\tau ,v,D v^m\big ) = {{\,\mathrm{div}\,}}\widehat{F} \qquad \text{ in }\, B_{4\varrho }\times (0,\tau _0) \end{aligned}$$

with

$$\begin{aligned} \widehat{{\mathbf {A}}}(x,\tau ,v,\xi ) := \gamma (\tau ) \Phi '(\tau ) {\mathbf {A}}\left( x,\Phi (\tau ),\tfrac{v}{\gamma (\tau )}, \tfrac{\xi }{\gamma ^m(\tau )}\right) \end{aligned}$$

and

$$\begin{aligned} \widehat{F}(x,\tau ) := \gamma (\tau ) \Phi '(\tau ) F(x,\Phi (\tau )). \end{aligned}$$

Using the growth assumptions (1.4) of ${\mathbf {A}}$ together with the definition of the functions $\Phi$ and $\gamma$ we compute that $\widehat{{\mathbf {A}}}$ satisfies the growth and ellipticity conditions

$$\begin{aligned} \widehat{{\mathbf {A}}}(x,\tau ,u,\xi ) \cdot \xi \ge \tfrac{\nu }{2} |\xi |^p \quad \text { and } \quad |\widehat{{\mathbf {A}}}(x,\tau ,u,\xi )| \le \tfrac{L}{2} |\xi |^{p-1}. \end{aligned}$$

Defining

$$\begin{aligned} k_0 := \varepsilon \left( \delta \varrho ^p \right) ^\frac{1}{d}, \end{aligned}$$

we observe that $k_0=\varepsilon \gamma (\tau ) s(\tau ) M$ for any $\tau \in [0,\tau _0]$ and therefore inequality (6.9) can be rewritten as

6.10 $$\begin{aligned} \big |\{ v(\tau ) \ge k_0 \}\cap B_\varrho (x_o) \big | \ge \tfrac{\alpha }{2} |B_\varrho (x_o)| \qquad \text { for all } \tau \in [0,\tau _0]. \end{aligned}$$

### Gradient estimates on intrinsic sublevel sets

Next, we define

$$\begin{aligned} \vartheta := \left( \frac{2^{j_\star }}{k_0} \right) ^{d} = \frac{1}{\delta \varrho ^p} \left( \frac{2^{j_\star }}{\varepsilon } \right) ^{d} \end{aligned}$$

and consider cylindersfor $0<r \le 8\varrho$. Moreover, for $j=1,\ldots ,j_\star$, we let

$$\begin{aligned} k_j := 2^{-j}k_0, \quad A_j(\tau ) := \{v(\tau )<k_j\}\cap B_{4\varrho }(x_o), \quad A_j := \{ v < k_j \}\cap \widehat{Q}_{4\varrho }^\vartheta \end{aligned}$$

and observe that

$$\begin{aligned} |A_j| = \int _{\vartheta (2\varrho )^p}^{\vartheta (4\varrho )^p} |A_j (\tau )| \,\mathrm {d}\tau . \end{aligned}$$

Further, a simple computation shows thatBy definition of $\widehat{F}$ and Hölder’s inequality, we obtain for $q\in [1,\sigma ]$ thatwhere in the second last line we used the area formula and the fact that $\vartheta (4\varrho )^p=\frac{4^p}{\delta }(\frac{2^{j_\star }}{\varepsilon })^{d}$, so that $c=c(n,m,p,\nu ,L,\sigma ,\alpha ,j_\star )$. Assuming that

6.11 $$\begin{aligned} \Vert F \Vert _{L^\sigma (\Omega _T)} \le \tfrac{1}{c_\star } \big (\tfrac{M^{m}}{\varrho }\big )^{p-1} |Q_{\varrho , M^{-d}\varrho ^p}^+(z_o)|^{\frac{1}{\sigma }} \end{aligned}$$

for some constant $c_\star \ge 1$ to be chosen later, we further estimateagain with a constant $c=c(n,m,p,\nu ,L,\sigma ,\alpha ,j_\star )$. Therefore, we may choose $c_\star$ in dependence on $n,m,p,\nu ,L,\sigma ,\alpha$ and $j_\star$ in such a way that6.12holds true for any $q\in [1,\sigma ]$. Note that we replaced $\varrho$ by $2\varrho$ in the denominator and  by  for later purpose. We observe thatThus, the Caccioppoli inequality (3.1) from Lemma 3.1 together with Lemma 2.7 (i), estimate (6.12) with $q=\frac{p}{p-1}$ and the fact that  implies6.13with $c=c(n,m,p,\nu ,L)$.

### Measure estimates for intrinsic sublevel sets

Now, we exploit the estimate

$$\begin{aligned} k_j^m - k_{j+1}^m = \left( 2^{-jm} k_0^m - 2^{-(j+1)m}k_0^m\right) = 2^{-jm} \left( 1 - 2^{-m}\right) k_0^m \ge c(m) k_j^m \end{aligned}$$

with $c(m) \in (0,1)$ together with Lemma 2.5 and inequality (6.10) to obtain

$$\begin{aligned} c(m) k_j^m |A_{j+1}(\tau )|&\le \left( k_j^m-k_{j+1}^m\right) |A_{j+1}(\tau )| \\&\le \frac{c(n) \varrho ^{n+1}}{|B_{4\varrho }(x_o)\setminus A_{j}(\tau )|} \int _{B_{4\varrho }(x_o) \cap \{k_{j+1}^m< v^m(\tau ) < k_j^m\}} |D v^m(\tau )| \,\mathrm {d}x\\&\le \tfrac{c(n) \varrho }{\alpha } \int _{A_j(\tau )\setminus A_{j+1}(\tau )} |D v^m(\tau )| \,\mathrm {d}x \end{aligned}$$

for all $j=0,\ldots ,j_\star$ and all $\tau \in (0, \vartheta (4\varrho )^p)$. We integrate this inequality with respect to $\tau$ over $(\vartheta (2\varrho )^p, \vartheta (4\varrho )^p)$, apply Hölder’s inequality on the right-hand side and use the gradient bound (6.13) to get

$$\begin{aligned} k_j^m|A_{j+1}|&\le \frac{c(n,m) \varrho }{\alpha } \int _{A_j\setminus A_{j+1}} |D v^m| \,\mathrm {d}x\mathrm {d}\tau \\&\le \frac{c(n,m) \varrho }{\alpha } \bigg ( \int _{A_j}|D v^m|^p \,\mathrm {d}x\mathrm {d}\tau \bigg )^{\frac{1}{p}} |A_j\setminus A_{j+1}|^{\frac{p-1}{p}} \\&\le \tfrac{c(n,m,p,\nu ,L)}{\alpha }\, k_j^m |A_j\setminus A_{j+1}|^{\frac{p-1}{p}} |\widehat{Q}_{4\varrho }^\vartheta |^{\frac{1}{p}}. \end{aligned}$$

Dividing both sides by $k_j^m > 0$ and summing over $j=0,\dots ,j_\star -1$, we find that

$$\begin{aligned} j_\star |A_{j_\star }|^\frac{p}{p-1} \le \sum _{j=1}^{j_\star } |A_j|^\frac{p}{p-1} \le \frac{c}{\alpha ^\frac{p}{p-1}}\, |\widehat{Q}_{4\varrho }^\vartheta |^{\frac{1}{p-1}} \sum _{j=0}^{j_\star -1} |A_j \setminus A_{j+1}| \le \frac{c}{\alpha ^\frac{p}{p-1}}\, |\widehat{Q}_{4\varrho }^\vartheta |^{\frac{p}{p-1}}, \end{aligned}$$

so that

6.14 $$\begin{aligned} \big |\{ v < k_{j_\star } \}\cap \widehat{Q}_{4\varrho }^\vartheta \big | = |A_{j_\star }| \le \alpha ^{-1}\big (\tfrac{c}{j_\star }\big )^\frac{p-1}{p} |\widehat{Q}_{4\varrho }^\vartheta | \end{aligned}$$

for a constant *c* depending only on *n*, *m*, *p*, $\nu$ and *L*.

### Application of De Giorgi type lemma 5.1

At this stage, we exploit Lemma 5.1. Observe that the cylinder $\widehat{Q}_{4\varrho }^\vartheta = Q_{4\varrho ,\vartheta (4^p-2^p)\varrho ^p}^-(x_o,\vartheta (4\varrho )^p)$ satisfies the requirements of the Lemma with $\varrho$, $\theta$ and *M* replaced by $2\varrho$, $\frac{4^p-2^p}{4^p}$ and $k_{j_\star }$. Then, the constant $\nu _1$ from Lemma 5.1 depends only on *n*, *m*, *p*, $\nu$, *L* and $\sigma$, but is independent of $j_\star$. Note that (5.1) is implied by (6.12) applied with $q=\sigma$. Thus, choosing $j_\star$ large enough, so that

$$\begin{aligned} \left( \tfrac{c}{j_\star }\right) ^\frac{p-1}{p} \le \alpha \nu _1, \end{aligned}$$

all assumptions of Lemma 5.1 are satisfied and we conclude that

6.15 $$\begin{aligned} v \ge \tfrac{1}{2} k_{j_\star } \qquad \text {a.e.~in } B_{2\varrho }(x_o) \times \left( \left( 4^p-2^p+1\right) \vartheta \varrho ^p, \vartheta (4\varrho )^p \right) . \end{aligned}$$

Note that $j_\star$ depends on $n,m,p,\nu ,L,\sigma$ and $\alpha$. This also fixes $c_\star$ in (6.11) in dependence on $n,m,p,\nu ,L,\sigma$ and $\alpha$. In turn, we choose $c \ge 1$ in dependence on $n,m,p,\nu ,L,\sigma$ and $\alpha$ in such a way that condition (6.7) implies the validity of (6.11) and (6.8).

### Returning to the original problem and conclusion

Finally we use the definition of *v* and $k_0$ to rewrite (6.15) as

$$\begin{aligned} u(x,\Phi (\tau )) \ge 2^{-(j_\star +1)} e^{-\frac{\tau }{d}}\varepsilon M \ge \kappa M \end{aligned}$$

for a.e. $$(x,\tau )\in B_{2\varrho } \times \left( (4^p-2^p+1) \vartheta \varrho ^p, \vartheta (4\varrho )^p \right] $$, where

$$\begin{aligned} \kappa = \kappa (n,m,p,\nu ,L,\alpha ) := 2^{-(j_\star +1)}\varepsilon e^{-\frac{\vartheta (4\varrho )^p}{d}}. \end{aligned}$$

Returning to the original time variable, we obtain

$$\begin{aligned} u \ge \kappa M \qquad \text {a.e.~in } B_{2\varrho } \times \big (t_o+\beta b (\kappa M)^{-d}\varrho ^p, t_o+b(\kappa M)^{-d}\varrho ^p\big ] \end{aligned}$$

with $b := \delta \varepsilon ^d2^{-(j_\star +1)d} \in (0,1)$ and $\beta := e^{-(2^p-1) \vartheta \varrho ^p}$ depending only on the data. Note that by the definitions of *b* and $\kappa$ we have $\frac{1}{\delta }\exp \big (-\frac{2^{2p+j_\star d}}{\delta \varepsilon ^{d}}\big )=\frac{\kappa ^d}{b}$, so that $\varrho _0$ can be re-written exactly as in (6.5). Since $\beta \le \frac{1}{2}$ this completes the proof of Proposition 6.3. $\square$

#### Remark 6.4

From the proof of Proposition 6.3 we observe that

$$\begin{aligned} \frac{b}{\kappa ^d} = \delta \exp \left( \frac{4^p}{\delta }\left( \frac{2^{j_\star }}{\varepsilon }\right) ^d\right)>4^p\delta \exp \left( \tfrac{1}{\delta }\right) >4^p. \end{aligned}$$

Moreover, the parameter *b* in Proposition 6.3 is monotonically increasing with respect to $\alpha$. This can be seen from the definition $b= \delta \varepsilon ^d2^{-(j_\star +1)d}$, where $j_\star$ is decreasing and $\varepsilon$ and $\delta$ are increasing with respect to $\alpha$; see Remark 6.2.

## Harnack’s inequality

We are now ready to prove our main result, Theorem 1.2. In the following section, the second (forward in time) inequality of (1.8) is shown. In a subsequent step, we ensure the validity of the first (backward in time) inequality of (1.8).

### Forward inequality

Let $c_o \ge 1$ to be fixed later, consider $(x_o,t_o) \in \Omega _T$ with $u(x_o,t_o)>0$ and define

$$\begin{aligned} \theta = \left( \frac{c_o}{u(x_o,t_o)} \right) ^{d}. \end{aligned}$$

Moreover, assume that $\varrho > 0$ is small enough so that $B_{9\varrho }(x_o) \times (t_o - 2\theta \varrho ^p , t_o + 2\theta \varrho ^p) \Subset \Omega _T$. Note that the stronger assumption $B_{9\varrho }(x_o) \times (t_o - 4\theta \varrho ^p , t_o + 4\theta \varrho ^p) \Subset \Omega _T$ will only be needed in the proof of the backward Harnack inequality. Finally, we define the rescaled function

7.1 $$\begin{aligned} v(x,t) := \tfrac{1}{u(x_o,t_o)} u\left( \tilde{x}(x),\tilde{t}(t)\right) \quad \text { in } B_9(0) \times \left( -2 c_o^{d}, 2 c_o^{d}\right) , \end{aligned}$$

where $(\tilde{x},\tilde{t}) :\widehat{\Omega _T} \rightarrow \Omega _T$ with $\widehat{\Omega _T} := \{(x,t) \in {\mathbb {R}}^{n+1}: (\tilde{x},\tilde{t}) \in \Omega _T\}$ is defined by

$$\begin{aligned} \tilde{x}(x):= x_o + \varrho x \quad \text { and } \quad \tilde{t}(t):= t_o + \frac{t \varrho ^p}{u(x_o,t_o)^{d}}. \end{aligned}$$

A straightforward computation shows that *v* is a bounded, continuous, non-negative weak super-solution of

$$\begin{aligned} \partial _t v - {{\,\mathrm{div}\,}}\tilde{{\mathbf {A}}}\left( x,t,v,Dv^m\right) = {{\,\mathrm{div}\,}}\tilde{F} \end{aligned}$$

in $B_9(0) \times (-2 c_o^{d}, 2 c_o^{d})$ in the sense of Definition 1.1 with

$$\begin{aligned} \tilde{{\mathbf {A}}}(x,t,v,\zeta ) = \frac{\varrho ^{p-1}}{u\left( x_o,t_o\right) ^{d+1}}\, {\mathbf {A}}\left( \tilde{x}, \tilde{t}, u(x_o,t_o) v, \tfrac{u\left( x_o,t_o\right) ^m}{\varrho } \zeta \right) \end{aligned}$$

and

$$\begin{aligned} \tilde{F}(x,t) = \frac{\varrho ^{p-1}}{u(x_o,t_o)^{d+1}}\, F(\tilde{x}, \tilde{t}). \end{aligned}$$

The main step towards Theorem 1.2 is the following lemma. After returning to the original variables this proves the intrinsic forward Harnack inequality, i.e. the second inequality of (1.8). Indeed, if Lemma 7.1 is valid, we obtain that

$$\begin{aligned} \Vert F \Vert _{L^\sigma (\Omega _T)} \ge \gamma _0 |B_1(0)|^{\frac{1}{\sigma }} \varrho ^{\frac{n+p}{\sigma }-(p-1)} u(x_o,t_o)^{m(p-1)-\frac{d}{\sigma }} \end{aligned}$$

or

$$\begin{aligned} u\left( x_o,t_o\right) \le \tfrac{1}{\gamma _1} u\left( \cdot ,t_o+ \theta \varrho ^p \right) \quad \text { in } B_\varrho (x_o), \end{aligned}$$

which shows the second inequality of (1.8) for $\gamma =\max \big \{\frac{1}{\gamma _0|B_1(0)|^{1/\sigma }}, \frac{1}{\gamma _1}\big \}$.

#### Lemma 7.1

For *v*, $\tilde{{\mathbf {A}}}$ and $\tilde{F}$ as above, there exist constants $\gamma _0, \gamma _1 \in (0,1)$ and $c_o > 1$ depending only on the data, but independent of $u(x_o,t_o)$ such that either

$$\begin{aligned} \Vert \tilde{F} \Vert _{L^\sigma \left( \widehat{\Omega _T}\right) } \ge \gamma _0 |B_1(0)|^{\frac{1}{\sigma }} \end{aligned}$$

or

$$\begin{aligned} v \left( \cdot ,c_o^d \right) \ge \gamma _1 \qquad \text { in } B_1(0). \end{aligned}$$

#### Proof

In the following we abbreviate $Q^-_r:=Q^-_{r,r^p}(0)=B_r(0)\times (-r^p,0]$ for $r>0$. For $\tau \in [0,1)$ we consider the family of cylinders $\{ Q_\tau ^- \}$ and the functions $M,N :[0,1) \rightarrow [0,\infty )$ defined by

$$\begin{aligned} M(\tau ) := \sup _{Q_\tau ^-} v, \qquad N(\tau ) := (1-\tau )^{-\delta }, \end{aligned}$$

with $\delta >1$ to be chosen later on. Note that the functions *M* and *N* are both monotonically increasing and $M_0 = 1 = N_0$, since $v(0,0)=1$. Moreover, as $\tau \uparrow 1$, $N(\tau ) \rightarrow \infty$ while $M(\tau )$ remains bounded, since *v* is bounded in $Q_1^-$. Together with the continuity of *v* this ensures that there exist

$$\begin{aligned} \tau _\star := \max \{ \tau \in [0,1) : M(\tau ) = N(\tau ) \} \end{aligned}$$

and $(x_\star ,t_\star ) \in Q_{\tau _\star }^-$ such that

7.2 $$\begin{aligned} v\left( x_\star ,t_\star \right) = M\left( \tau _\star \right) = N\left( \tau _\star \right) = \left( 1-\tau _\star \right) ^{-\delta }. \end{aligned}$$

Let $\tilde{n} \in {\mathbb {N}}_{\ge 2}$ such that $2^{1-\tilde{n}} < 1- \tau _\star \le 2^{2-\tilde{n}}$ and define $r:=2^{-\tilde{n}}$. Then $\tau _\star + r < \tau _\star +\frac{1}{2}(1-\tau _\star ) =\frac{1+\tau _\star }{2}$, which implies

$$\begin{aligned} \left( x_\star ,t_\star \right) +Q_r^- \subset Q_{\frac{1+\tau _\star }{2}}^- \subset Q_1^-. \end{aligned}$$

Moreover, by definition of *M*, *N* and $\tau _\star$ we have

$$\begin{aligned} \sup _{\left( x_\star ,t_\star \right) +Q_r^-} v \le \sup _{Q_{\frac{1+\tau _\star }{2}}^-} v = M\left( \tfrac{1+\tau _\star }{2}\right) \le N\left( \tfrac{1+\tau _\star }{2}\right) = \left( \tfrac{1-\tau _\star }{2}\right) ^{-\delta } \le 2^{\tilde{n}\delta }=r^{-\delta } =: M_\star . \end{aligned}$$

Observe that $M_\star >1$. Next, on the cylinder $Q^-_{r,M_\star ^{-d} r^p}(x_\star , t_\star ) \subset (x_\star ,t_\star )+Q_r^-$ we apply the De Giorgi type Lemma 5.2 to *v* with

$$\begin{aligned} \zeta =1-2^{-4\delta m}, \qquad a=\frac{1-2^{-3 \delta m}}{1-2^{-4 \delta m}} \end{aligned}$$

and $(\mu _+,M,\theta ,\varrho )$ replaced by $(M_\star ,M_\star ,1,\tfrac{r}{2})$. Indeed, hypothesis (5.2) is satisfied, since

$$\begin{aligned} \sup _{Q^-_{r,M_\star ^{-d} r^p}\left( x_\star , t_\star \right) } v \le \sup _{\left( x_\star ,t_\star \right) +Q_r^-} v \le M_\star . \end{aligned}$$

By ${\tilde{\nu }}$ we denote the constant $\nu _2$ from Lemma 5.2 depending on $n,m,p,\nu ,L,\theta ,a,\zeta$; hence ${\tilde{\nu }}={\tilde{\nu }}(n,m,p,\nu ,L,\delta )$. Moreover, observe that

$$\begin{aligned} v^m\left( x_\star ,t_\star \right)&= \left( 1-\tau _\star \right) ^{-\delta m} \ge 2^{-2 \delta m} r^{-\delta m} = 2^{-2\delta m} M_\star ^m >M_\star ^m - a \zeta M_\star ^m. \end{aligned}$$

This shows that conclusion (5.5) of Lemma 5.2 is false. Hence, either (5.3) or (5.4) is violated. This means, we either have

7.3 $$\begin{aligned} \Vert \tilde{F} \Vert _{L^\sigma \left( \widehat{\Omega _T}\right) } >\left( \frac{2M_\star ^m}{r}\right) ^{p-1} \big | Q^-_{r,M_\star ^{-d} r^p}\left( x_\star , t_\star \right) \big |^{\frac{1}{\sigma }} \end{aligned}$$

or

7.4 $$\begin{aligned} \big | \big \{ v \ge 2^{-4\delta } M_\star \big \} \cap Q^-_{r,M_\star ^{-d} r^p}\left( x_\star , t_\star \right) \big | >{\tilde{\nu }} \big | Q^-_{r,M_\star ^{-d} r^p}\left( x_\star , t_\star \right) \big |. \end{aligned}$$

If (7.4) is satisfied, by Fubini’s theorem there exists ${\bar{t}}_\star \in (t_\star -M_\star ^{-d}r^p,t_\star ]$ with

$$\begin{aligned} | \{ v\left( {\bar{t}}_\star \right) \ge 2^{-4\delta } M_\star \} \cap B_r\left( x_\star \right) | > {\tilde{\nu }} |B_r\left( x_\star \right) |. \end{aligned}$$

By $\tilde{b}, {\tilde{\kappa }}\in (0,1)$ and $\tilde{c}\ge 1$ we denote the constants $b,\kappa ,c$ from the Expansion of Positivity in Proposition 6.3 applied with $\alpha ={{\tilde{\nu }}}$. Note that $\tilde{b}, {\tilde{\kappa }}$ and $\tilde{c}$ depend on $n, m, p, \nu , L,\sigma$ and $\delta$. Supposed that

7.5 $$\begin{aligned} r \le \min \bigg \{\tfrac{1}{8}{{\,\mathrm{dist}\,}}\left( x_\star ,\partial B_9(0)\right) , \left[ \frac{\left( 2 c_o^d-{{\bar{t}}}_\star \right) \left( 2^{-4\delta } {{\tilde{\kappa }}} M_\star \right) ^d}{{\tilde{b}}}\right] ^{\frac{1}{p}}\bigg \}, \end{aligned}$$

we are allowed to apply Proposition 6.3 with $(F,\alpha ,M,\varrho )$ replaced by $(\tilde{F},{\tilde{\nu }},2^{-4\delta } M_\star ,r)$ and conclude that either

7.6 $$\begin{aligned} \Vert \tilde{F} \Vert _{L^\sigma \left( \widehat{\Omega _T}\right) } \ge \frac{1}{\tilde{c}} \left( \frac{\left( 2^{-4\delta } M_\star \right) ^m}{r}\right) ^{p-1} \big |Q^+_{r,\left( 2^{-4\delta } M_\star \right) ^{-d} r^p}\big |^{\frac{1}{\sigma }} \end{aligned}$$

or

$$\begin{aligned} v \ge 2^{-4\delta } {\tilde{\kappa }} M_\star \quad \text{ in }\, B_{2r}\left( x_\star \right) \times \big ({\bar{t}}_\star +\tfrac{1}{2} \tilde{b} \big (2^{-4\delta }{{\tilde{\kappa }}} M_\star \big )^{-d} r^p, {\bar{t}}_\star +\tilde{b} \big (2^{-4\delta }{{\tilde{\kappa }}} M_\star \big )^{-d} r^p\big ] \end{aligned}$$

holds true. In the second case we find that

7.7 $$\begin{aligned} \big |\{ v(\tilde{t}_o) \ge 2^{-4\delta } {\tilde{\kappa }} M_\star \} \cap B_{2r}(x_\star )\big | = | B_{2r}(x_\star ) |, \end{aligned}$$

where $\tilde{t}_o := {\bar{t}}_\star + \tilde{b} \big ( 2^{-4\delta } {{\tilde{\kappa }}} M_\star \big )^{-d} r^p$. This allows to apply the Expansion of Positivity in the next step with $\alpha =1$. Therefore, by $b, \kappa \in (0,1)$ and $c\ge 1$ we denote the constants $b,\kappa ,c$ from Proposition 6.3 applied with $\alpha =1$. Then, $b, \kappa$ and *c* depend on $n, m, p, \nu , L$ and $\sigma$, but not on $\delta$. Supposed that

7.8 $$\begin{aligned} 2r \le \min \bigg \{\tfrac{1}{8}{{\,\mathrm{dist}\,}}\left( x_\star ,\partial B_9(0)\right) , \bigg [\frac{(2 c_o^d-{\tilde{t}}_o)\left( 2^{-4\delta } {{\tilde{\kappa }}}\kappa M_\star \right) ^d}{b}\bigg ]^{\frac{1}{p}}\bigg \}, \end{aligned}$$

we may apply Proposition 6.3 with $(F,\alpha ,M,\varrho )$ replaced by $(\tilde{F},1,2^{-4\delta } {\tilde{\kappa }}M_\star ,2r)$ and conclude that either

7.9 $$\begin{aligned} \Vert \tilde{F} \Vert _{L^\sigma \left( \widehat{\Omega _T}\right) } \ge \frac{1}{c} \left( \frac{\left( 2^{-4\delta }{{\tilde{\kappa }}} M_\star \right) ^m}{2r}\right) ^{p-1} \big |Q^+_{2r,\left( 2^{-4\delta } {{\tilde{\kappa }}} M_\star \right) ^{-d} (2r)^p}\big |^{\frac{1}{\sigma }} \end{aligned}$$

or

$$\begin{aligned} v \ge 2^{-4\delta } {\tilde{\kappa }} \kappa M_\star \quad \text { in } B_{4r}(x_\star ) \times \big (t_1-\tfrac{1}{2} b \left( 2^{-4\delta }{\tilde{\kappa }}\kappa M_\star \right) ^{-d} (2r)^p, t_1\big ] \end{aligned}$$

holds true, where $t_1 := {\tilde{t}}_o + b \big (2^{-4\delta } {\tilde{\kappa }}\kappa M_\star \big )^{-d} (2r)^p$. In the second case, we have

$$\begin{aligned} \big |\{ v(t_1) > 2^{-4\delta } {\tilde{\kappa }} \kappa M_\star \}\cap B_{4r}(x_\star ) \big | = | B_{4r}(x_\star ) |. \end{aligned}$$

We recursively define $t_2,\ldots ,t_{\tilde{n}}$ by

$$\begin{aligned} t_j := t_{j-1} + b \big (2^{-4\delta } {\tilde{\kappa }} \kappa ^{j} M_\star \big )^{-d} (2^j r)^p \end{aligned}$$

for $j \in \{2,\ldots ,\tilde{n}\}$. Iterating the procedure of Expansion of Positivity we arrive at the following assertion. Supposed that

7.10 $$\begin{aligned} 2^j r \le \min \bigg \{\tfrac{1}{8}{{\,\mathrm{dist}\,}}(x_\star ,\partial B_9(0)), \bigg [\frac{(2 c_o^d- t_{j-1})(2^{-4\delta } {{\tilde{\kappa }}}\kappa ^j M_\star )^d}{b}\bigg ]^{\frac{1}{p}}\bigg \}, \end{aligned}$$

for every $j=2,\ldots ,\tilde{n}$, we find that either

7.11 $$\begin{aligned} \Vert \tilde{F} \Vert _{L^\sigma (\widehat{\Omega _T})} > \frac{1}{c} \bigg (\frac{(2^{-4\delta } {\tilde{\kappa }} \kappa ^{j-1} M_\star )^m}{2^{j}r}\bigg )^{p-1} \big |Q^+_{2^{j}r, (2^{-4\delta } {\tilde{\kappa }} \kappa ^{j-1} M_\star )^{-d}(2^{j}r)^p}\big |^{\frac{1}{\sigma }} \end{aligned}$$

is satisfied for some $j\in \{2,\ldots ,\tilde{n}\}$ or

7.12 $$\begin{aligned} v \ge 2^{-4\delta } {\tilde{\kappa }} \kappa ^{\tilde{n}} M_\star \quad \text{ in } \,B_{2^{\tilde{n}+1}r}(x_\star ) \times \big (t_{\tilde{n}} -\tfrac{1}{2} b \big (2^{-4\delta }{\tilde{\kappa }}\kappa ^{\tilde{n}}M_\star \big )^{-d} (2^{\tilde{n}}r)^p, t_{\tilde{n}}\big ]. \end{aligned}$$

We first ensure that (7.10) is satisfied for ${\tilde{n}}$. We note that $2^{\tilde{n}}r=1$. Since $x_\star \in B_1(0)$, we immediately observe that $2^{\tilde{n}}r=1\le \tfrac{1}{8}{{\,\mathrm{dist}\,}}(x_\star ,\partial B_9(0))$. Next, we choose $\delta > 1$ in dependence on $n,m,p,\nu ,L$ and $\sigma$ such that $2^\delta \kappa =1$, which is possible, since $\kappa$ is independent of $\delta$. In view of the definition of $M_\star$ we find that

$$\begin{aligned} 2^{-4\delta } {\tilde{\kappa }} \kappa ^{\tilde{n}} M_\star = 2^{-4\delta } {\tilde{\kappa }} \kappa ^{\tilde{n}} 2^{\tilde{n}\delta } = 2^{-4\delta } {\tilde{\kappa }} ( 2^{\delta } \kappa )^{\tilde{n}} = 2^{-4 \delta } {\tilde{\kappa }} =: \gamma _1 \in (0,1). \end{aligned}$$

Note that $\gamma _1$ depends on $n,m,p,\nu ,L$ and $\sigma$. The second condition in (7.10) is equivalent to $t_{{\tilde{n}}}\le 2 c_o^d$. Therefore, we compute

$$\begin{aligned} t_{\tilde{n}}&= {\bar{t}}_\star + \tilde{b} \big (2^{-4\delta } {\tilde{\kappa }} M_\star \big )^{-d} r^p + b \sum _{j=1}^{\tilde{n}} \left( 2^{-4\delta } {\tilde{\kappa }} \kappa ^{j} M_\star \right) ^{-d} \left( 2^j r\right) ^p \\&= {\bar{t}}_\star + \frac{\tilde{b}}{\gamma _1^{d}} \bigg (\frac{\kappa ^{d}}{2^p}\bigg )^{\tilde{n}} + \frac{2^p b}{\gamma _1^{d}(2^p-\kappa ^d)} \bigg [1-\bigg (\frac{\kappa ^{d}}{2^p}\bigg )^{\tilde{n}}\bigg ] \\&= {\bar{t}}_\star + \frac{2^p b}{\gamma _1^{d}(2^p-\kappa ^d)} - \frac{1}{\gamma _1^{d}} \bigg [\frac{2^p b}{2^p-\kappa ^d} - \tilde{b} \bigg ] \bigg (\frac{\kappa ^{d}}{2^p}\bigg )^{\tilde{n}} . \end{aligned}$$

We note that due to Remark 6.4 we have ${\tilde{b}}\le b$ and therefore the expression $\frac{2^p b}{2^p-\kappa ^d} - \tilde{b}$ is positive. Hence, choosing $c_o$ such that

7.13 $$\begin{aligned} 2 c_o^d\ge \frac{1}{\gamma _1^d} \frac{2^p b}{(2^p-\kappa ^d)} \end{aligned}$$

and taking into account that ${\bar{t}}_\star \le 0$ we find that

$$\begin{aligned} t_{\tilde{n}} \le {\bar{t}}_\star + 2 c_o^d\le 2 c_o^d. \end{aligned}$$

Provided that (7.13) holds true, (7.10) is satisfied for ${\tilde{n}}$ and in turn implies that (7.10) is satisfied for any $j=2,\ldots ,\tilde{n}$ and in particular also (7.5) and (7.8) are satisfied.

To summarize, we have now shown that either (7.12) is satisfied or one of the alternatives (7.6), (7.9) or (7.11) if $c_o$ is chosen large enough. We start with the former case where (7.12) is satisfied. Since $2^{\tilde{n}}r=1$ we have $B_1(0) \subset B_2(x_\star ) = B_{2^{\tilde{n}+1}r}(x_\star )$, so that

7.14 $$\begin{aligned} v(t) \ge \gamma _1 \quad \text { in}\, B_1(0) \text { for any}\, t \in \big (t_{\tilde{n}}-\tfrac{1}{2} b \gamma _1^{-d}, t_{\tilde{n}}\big ]. \end{aligned}$$

Unfortunately, the interval depends on $\tilde{n}$ and hence on *v*. Therefore, we need to find a subinterval which is independent of $\tilde{n}$. In view of Remark 6.4 we have $\frac{b}{{{\tilde{\kappa }}}^d}\ge \frac{{\tilde{b}}}{{{\tilde{\kappa }}}^d}>4^{p}$ and hence $\gamma _1^d=2^{-4 \delta d} {\tilde{\kappa }}^d<2^{-2p-4 \delta d} b<2^{-2p} b$. Therefore, we observe from the preceding computation of $t_{{\tilde{n}}}$ that

$$\begin{aligned} t_{\tilde{n}}&\ge {\bar{t}}_\star + \frac{2^p b}{\gamma _1^{d}\left( 2^p-\kappa ^d\right) } \bigg [1-\bigg (\frac{\kappa ^{d}}{2^p}\bigg )^{\tilde{n}}\bigg ] \ge -1 + \frac{2^p b}{\gamma _1^{d}(2^p-\kappa ^d)} \bigg [1-\bigg (\frac{\kappa ^{d}}{2^p}\bigg )^{2}\bigg ] \\&> \frac{2^p b}{\gamma _1^{d}(2^p-\kappa ^d)} - \frac{\kappa ^{2d} b}{2^p\gamma _1^{d}(2^p-\kappa ^d)} - \frac{b}{2^{2p}\gamma _1^d} . \end{aligned}$$

In the second term on the right-hand side we use $\kappa< 4^{-p}b<4^{-p}$, which once again is a consequence of Remark 6.4. This leads us to the lower bound

$$\begin{aligned} t_{\tilde{n}}> \frac{2^p b}{\gamma _1^{d}(2^p-\kappa ^d)} - \frac{b}{2^{3p}\gamma _1^{d}} - \frac{b}{2^{2p}\gamma _1^d} > \frac{2^p b}{\gamma _1^{d}(2^p-\kappa ^d)} - \frac{b}{2^{2p-1}\gamma _1^{d}}. \end{aligned}$$

For the left interval limit in (7.14) we obtain

$$\begin{aligned} t_{\tilde{n}}-\tfrac{1}{2} b \gamma _1^{-d} \le \frac{2^p b}{\gamma _1^{d}(2^p-\kappa ^d)} - \frac{b}{2\gamma _1^{d}}. \end{aligned}$$

The preceding computations show that with the choice

$$\begin{aligned} c_o^d:= \frac{2^p b}{\gamma _1^{d}(2^p-\kappa ^d)} - \frac{b}{2^{p}\gamma _1^{d}}>1 \end{aligned}$$

we have

$$\begin{aligned} c_o^d\in \big (t_{\tilde{n}}-\tfrac{1}{2} b \gamma _1^{-d}, t_{\tilde{n}}\big ]. \end{aligned}$$

Note that $c_o$ depends on $n,m,p,\nu ,L$ and $\sigma$ and a straightforward calculation shows that (7.13) is satisfied. From (7.14) we now conclude that

$$\begin{aligned} v\left( c_o^d\right) \ge \gamma _1 \qquad \text { in}\, B_1(0), \end{aligned}$$

which concludes the proof of the lemma in the case that (7.12) is satisfied.

Finally, we are left with the case where one of the alternatives (7.6), (7.9) or (7.11) is satisfied. In any of these cases we conclude that

7.15 $$\begin{aligned} \Vert \tilde{F} \Vert _{L^\sigma \left( \widehat{\Omega _T}\right) } \ge \gamma _0 |B_1(0)|^{\frac{1}{\sigma }}, \qquad \text{ where } \gamma _0 := \frac{1}{\max \{c,\tilde{c}\}} \left( \frac{{{\tilde{\kappa }}}}{2^{4\delta }\kappa }\right) ^{d+1-\frac{d}{\sigma }} \end{aligned}$$

is valid. We note that $\gamma _0\in (0,1)$, since ${{\tilde{\kappa }}}\le \kappa$ by Remark 6.4 and that $\gamma _0$ depends on $n,m,p,\nu ,L$ and $\sigma$. This concludes the proof of the lemma. $\square$

### Backward inequality

With the intrinsic forward Harnack inequality on hand, we are able to show the intrinsic backward Harnack inequality, i.e. the first inequality of (1.8). Actually, in the following we will prove the more general version

7.16 $$\begin{aligned} \left( c_1\gamma ^{c_2}\right) ^{-1} \sup _{B_\varrho (x_o)} u\left( \cdot ,t_o-\left( c_1 \gamma ^{c_2-1}\right) ^{-d}\theta \varrho ^p\right) \le u\left( x_o,t_o\right) \end{aligned}$$

with positive constants $c_1,c_2$ such that $c_1\gamma ^{c_2-2} > 1$, which implies the first inequality of (1.8) by choosing $c_1 = c_2 = 2$. We have already fixed $(x_o,t_o) \in \Omega _T$ with $u(x_o,t_o)>0$. Now we assume that $B_{9\varrho }(x_o) \times (t_o - 4\theta \varrho ^p, t_o + 4 \theta \varrho ^p) \Subset \Omega _T$. Moreover, let $c_1,c_2>0$ be positive constants such that $c_1\gamma ^{c_2-2}>1$ and suppose that alternative (1.7) is not valid, i.e.

7.17 $$\begin{aligned} \Vert F \Vert _{L^\sigma (\Omega _T)} \varrho ^{p-1-\frac{n+p}{\sigma }} < \tfrac{1}{\gamma }u(x_o,t_o)^{d+1-\frac{d}{\sigma }}. \end{aligned}$$

In order to prove the backward Harnack inequality, we consider two alternatives. First, we assume that

7.18 $$\begin{aligned} u\left( x_o,t\right) < c_1 \gamma ^{c_2-1} u\left( x_o,t_o\right) \quad \text { for all } t\in \left( t_o - 2 \theta \varrho ^p,t_o\right) \end{aligned}$$

with $\gamma$ as in the right-hand side of (1.8). Our aim is to prove that (7.18) implies

7.19 $$\begin{aligned} \sup _{B_\varrho (x_o)} u\left( \cdot , t_o-\left( c_1\gamma ^{c_2-1}\right) ^{-d}\theta \varrho ^p\right) < c_1 \gamma ^{c_2} u\left( x_o,t_o\right) . \end{aligned}$$

Indeed, assume that (7.19) was not satisfied. Then there exists $x_\star \in B_\varrho (x_o)$ such that $u(x_\star ,t_1) = c_1\gamma ^{c_2} u(x_o,t_o)$, where we abbreviated $t_1 := t_o-(c_1\gamma ^{c_2-1})^{-d}\theta \varrho ^p$, since *u* is continuous and (7.18) is in force. Let $\theta _\star := c_o^{d}u(x_\star ,t_1)^{-d}$. A simple calculation shows that

$$\begin{aligned} \left\{ \begin{array}{l} t_1 - 2 \theta _\star \varrho ^p = t_o - \left( 1 + 2 \gamma ^{-d}\right) \left( c_1\gamma ^{c_2-1}\right) ^{-d} \theta \varrho ^p,\\ t_1 + 2 \theta _\star \varrho ^p = t_o - \left( 1- 2 \gamma ^{-d}\right) \left( c_1\gamma ^{c_2-1}\right) ^{-d} \theta \varrho ^p. \end{array} \right. \end{aligned}$$

Since $d>0$, $\gamma >1$ and $c_1\gamma ^{c_2-1}>1$, this implies $(t_1 - 2 \theta _\star \varrho ^p, t_1 + 2 \theta _\star \varrho ^p) \subset (t_o - 4 \theta \varrho ^p, t_o + 4 \theta \varrho ^p) \Subset \Omega _T$. Thus, we are able to apply the forward Harnack inequality with $(x_o,t_o)$ replaced by $(x_\star , t_1)$. This leads to

$$\begin{aligned} \Vert F \Vert _{L^\sigma (\Omega _T)} \varrho ^{p-1-\frac{n+p}{\sigma }} \ge \tfrac{1}{\gamma }u\left( x_\star , t_1\right) ^{d+1-\frac{d}{\sigma }} > \tfrac{1}{\gamma }u(x_o,t_o)^{d+1-\frac{d}{\sigma }}, \end{aligned}$$

which contradicts (7.17), or

$$\begin{aligned} u\left( x_\star ,t_1\right) \le \gamma \inf _{B_\varrho \left( x_\star \right) } u\left( \cdot , t_1 + \theta _\star \varrho ^p\right) . \end{aligned}$$

In view of (7.18) and the facts that $x_o \in B_\varrho (x_\star )$ and $t_1 + \theta _\star \varrho ^p < t_o$, this yields the contradiction

$$\begin{aligned} c_1 \gamma ^{c_2} u\left( x_o,t_o\right)&= u\left( x_\star , t_1\right) \le \gamma u\left( x_o,t_1 + \theta _\star \varrho ^p\right) < c_1\gamma ^{c_2} u\left( x_o,t_o\right) . \end{aligned}$$

Therefore (7.18) implies (7.19).

It remains to treat the case where (7.18) is violated. This means that there exists $t \in (t_o - 2\theta \varrho ^p,t_o)$ such that $u(x_o,t) = c_1\gamma ^{c_2-1} u(x_o,t_o)$. We define $\tau$ as the largest value with this property (note that *u* is continuous) and let

$$\begin{aligned} \theta _\tau := \left( \frac{c_o}{u(x_o,\tau )} \right) ^{d} = \left( c_1 \gamma ^{c_2-1}\right) ^{-d} \theta . \end{aligned}$$

We claim that

7.20 $$\begin{aligned} t_o-\tau >\theta _\tau \varrho ^p. \end{aligned}$$

Indeed, if (7.20) was not valid, there existed $0 < {\tilde{\varrho }} \le \varrho$ such that

$$\begin{aligned} t_o - \tau = \theta _\tau {\tilde{\varrho }}^p. \end{aligned}$$

Computing that $(\tau - 2 \theta _\tau {\tilde{\varrho }}^p, \tau + 2 \theta _\tau {\tilde{\varrho }}^p) \subset (t_o - 4 \theta \varrho ^p, t_o + 4 \theta \varrho ^p)$, we are allowed to apply the forward Harnack inequality with $(x_o,\tau )$ instead of $(x_o,t_o)$. This gives that either

$$\begin{aligned} \Vert F \Vert _{L^\sigma \left( \Omega _T\right) } {\tilde{\varrho }}^{p-1-\frac{n+p}{\sigma }} \ge \tfrac{1}{\gamma }u\left( x_o,\tau \right) ^{d+1-\frac{d}{\sigma }} >\tfrac{1}{\gamma }u\left( x_o,t_o\right) ^{d+1-\frac{d}{\sigma }}, \end{aligned}$$

or

$$\begin{aligned} c_1\gamma ^{c_2-1} u\left( x_o,t_o\right) = u\left( x_o,\tau \right) \le \gamma u\left( x_o, \tau + \theta _\tau {\tilde{\varrho }}^p\right) = \gamma u\left( x_o,t_o\right) . \end{aligned}$$

holds true. The former one contradicts (7.17), while the latter one contradicts $c_1\gamma ^{c_2-2}>1$. Therefore, (7.20) is valid. Next, we define

$$\begin{aligned} s = t_o - \theta _\tau \varrho ^p. \end{aligned}$$

By definition of $\tau$ and (7.20), we find that

$$\begin{aligned} \tau< s< t_o \qquad \text { and } \qquad u\left( x_o,s\right) < c_1 \gamma ^{c_2-1} u\left( x_o,t_o\right) . \end{aligned}$$

In the following we show by contradiction that

7.21 $$\begin{aligned} \sup _{B_\varrho \left( x_o\right) } u(y,s) < c_1 \gamma ^{c_2-1} u\left( x_o,t_o\right) . \end{aligned}$$

Indeed, otherwise by the continuity of *u* there existed $y \in B_\varrho (x_o)$ with $u(y,s) = c_1 \gamma ^{c_2-1} u(x_o,t_o)$. For $\theta _s := c_o^{d}u(y,s)^{-d}$ we have that $(s - 2 \theta _s \varrho ^p, s + 2 \theta _s \varrho ^p) \subset (t_o - 4 \theta \varrho ^p, t_o + 4 \theta \varrho ^p)$. Thus, applying the forward Harnack inequality with (*y*, *s*) instead of $(x_o,t_o)$ leads to

$$\begin{aligned} \Vert F \Vert _{L^\sigma \left( \Omega _T\right) } \varrho ^{p-1-\frac{n+p}{\sigma }} \ge \tfrac{1}{\gamma }u(y,s)^{d+1-\frac{d}{\sigma }} >\tfrac{1}{\gamma }u(x_o,t_o)^{d+1-\frac{d}{\sigma }}, \end{aligned}$$

which contradicts (7.17), or

$$\begin{aligned} u(y,s) \le \gamma \inf _{B_\varrho (y)} u\left( \cdot ,s+\theta _s \varrho ^p\right) . \end{aligned}$$

Since $s+\theta _s \varrho ^p = t_o$ and $y \in B_\varrho (x_o)$, we obtain the contradiction

$$\begin{aligned} c_1 \gamma ^{c_2-1} u\left( x_o,t_o\right) = u(y,s) \le \gamma u\left( x_o,t_o\right) . \end{aligned}$$

Therefore (7.21) is valid. Recalling the definition of *s*, we conclude that the desired backwards Harnack inequality is in force also in this case. This finishes the proof of inequality (7.16) and thus the proof of Theorem 1.2.

## References

[1] Acerbi, E., Fusco, N.: Regularity for minimizers of nonquadratic functionals: the case $$1<p<2$$. J. Math. Anal. Appl. **140**(1), 115–135 (1989)

[2] Bögelein V, Lukkari T, Scheven C (2017). Hölder regularity for degenerate parabolic obstacle problems. Ark. Mat..

[3] Bögelein V, Duzaar F, Kinnunen J, Scheven C (2020). Higher integrability for doubly nonlinear parabolic systems. J. Math. Pures Appl..

[4] Bögelein V, Duzaar F, Korte R, Scheven C (2019). The higher integrability of weak solutions of porous medium systems. Adv. Nonlinear Anal..

[5] Bögelein V, Duzaar F, Marcellini P (2013). Parabolic systems with p; q-growth: a variational approach. Arch. Ration. Mech. Anal..

[6] Bögelein V, Lehtelä P, Sturm S (2019). Regularity of weak solutions and supersolutions to the porous medium equation. Nonlinear Anal..

[7] DiBenedetto E (1988). Intrinsic Harnack type inequalities for solutions of certain degenerate parabolic equations. Arch. Ration. Mech. Anal..

[8] DiBenedetto, E.: Degenerate parabolic equations, Springer Science & Business Media (1993)

[9] DiBenedetto E, Gianazza U, Vespri V (2008). Harnack estimates for quasi-linear degenerate parabolic differential equations. Acta Math..

[10] DiBenedetto E, Gianazza U, Vespri V (2012). Harnack’s Inequality for Degenerate and Singular Parabolic Equations.

[11] Düzgün FG, Fornaro S, Vespri V (2015). Interior Harnack estimates: the state-of-the-art for quasilinear singular parabolic equations. Milan J. Math..

[12] Fornario S, Sosio M (2008). Intrinsic Harnack estimates for some doubly nonlinear degenerate parabolic equations. Adv. Diff. Equ..

[13] Fornario S, Sosio M, Vespri V (2015). Harnack type inequalities for some doubly nonlinear singular parabolic equations. Discr. Cont. Dyn. Syst. A.

[14] Gianazza U, Vespri V (2006). A Harnack inequality for solutions of doubly nonlinear parabolic equations. J. Appl. Funct. Anal..

[15] Giaquinta M, Modica G (1986). Remarks on the regularity of the minimizers of certain degenerate functionals. Manuscripta Math..

[16] Hadamard J (1954). Extension à l’èquation de la chaleur d’un théorème de A. Harnack. Rend. Circ. Mat. Palermo.

[17] Ivanov, A.V.: Hölder estimates for a natural class of equations of fast diffusion type, (Russian) Zap. Nauchn. Sem. S.-Peterburg. Otdel. Mat. Inst. Steklov. (POMI) 229, : Chisl. Metody i Voprosy Organ. Vychisl. **11**(29–62), 322 (1995)

[18] Ivanov AV (1998). Hölder estimates for a natural class of equations of fast diffusion type, translation in. J. Math. Sci. (New York).

[19] Ivanov, A.V.: Hölder estimates for equations of slow and normal diffusion type, (Russian) Zap. Nauchn. Sem. S.-Peterburg. Otdel. Mat. Inst. Steklov. (POMI) 215, : Differentsial’naya Geom. Gruppy Li i Mekh. **14**(130–136), 311 (1994)

[20] Ivanov AV (1997). Hölder estimates for equations of slow and normal diffusion type, translation in. J. Math. Sci. (New York).

[21] Kinnunen J, Lindqvist P (2006). Pointwise behaviour of semicontinuous supersolutions to a quasilinear parabolic equation. Ann. Mat. Pura Appl..

[22] Kinnunen J, Kuusi T (2007). Local behaviour of solutions to doubly nonlinear parabolic equations Math. Annals.

[23] Kuusi, T.: Harnack estimates for weak supersolutions to nonlinear degenerate parabolic equations. Ann. Sc. Norm. Super. Pisa Cl. Sci. (5) (4), 673–716 (2008)

[24] Li Q (2018). Weak Harnack estimates for supersolutions to doubly degenerate parabolic equations. Nonlinear Anal..

[25] Moser J (1961). On Harnack’s theorem for elliptic differential equations. Comm. Pure Appl. Math..

[26] Moser J (1964). A Harnack inequality for parabolic differential equations. Comm. Pure Appl. Math..

[27] Pini B (1954). Sulla soluzione generalizzata di Wiener per il primo problema di valori al contorno nel caso parabolico. Rend. Sem. Mat. Univ. Padova.

[28] Porzio MM, Vespri V (1993). Hölder estimates for local solutions of some doubly nonlinear degenerate parabolic equations. J. Diff. Equ..

[29] Serrin J (1964). Local behavior of solutions of quasi-linear equations. Acta Math..

[30] Singer T, Vestberg M (2019). Local boundedness of weak solutions to the diffusive wave approximation of the shallow water equations. J. Diff. Equ..

[31] Trudinger NS (1967). On Harnack type inequalities and their application to quasilinear elliptic equations. Comm. Pure Appl. Math..

[32] Trudinger NS (1968). Pointwise estimates and quasilinear parabolic equations. Comm. Pure Appl. Math..

[33] Vespri V (1994). Harnack type inequalities for solutions of certain doubly nonlinear parabolic equations. J. Math. Anal. Appl..

[34] Vespri, V., Vestberg, M.: An extensive study of the regularity of solutions to doubly singular equations. Adv. Calc. Var. (2020). 10.1515/acv-2019-0102

